# Identification of *KANSARL* as the first cancer predisposition fusion gene specific to the population of European ancestry origin

**DOI:** 10.18632/oncotarget.16385

**Published:** 2017-03-24

**Authors:** Jeff Xiwu Zhou, Xiaoyan Yang, Shunbin Ning, Ling Wang, Kesheng Wang, Yanbin Zhang, Fenghua Yuan, Fengli Li, David D. Zhuo, Liren Tang, Degen Zhuo

**Affiliations:** ^1^ Department of Medicine, School of Medicine, Ningbo University, Ningbo, China; ^2^ SplicingCodes.com, Biotailor Inc., Palmetto Bay, FL, USA; ^3^ Department of Internal Medicine, Quillen College of Medicine, East Tennessee State University, Johnson City, TN, USA; ^4^ Department of Biostatistics and Epidemiology, East Tennessee State University, Johnson City, TN, USA; ^5^ Department of Biochemistry and Molecular Biology, University of Miami, Miami, FL, USA

**Keywords:** *KANSARL*, fusion gene, European ancestry origin, RNA-seq

## Abstract

Gene fusion is one of the hallmarks of cancer. Recent advances in RNA-seq of cancer transcriptomes have facilitated the discovery of fusion transcripts. In this study, we report identification of a surprisingly large number of fusion transcripts, including six *KANSARL* (*KANSL1*-*ARL17A*) transcripts that resulted from the fusion between the *KANSL1* and *ARL17A* genes using a RNA splicingcode model. Five of these six *KANSARL* fusion transcripts are novel. By systematic analysis of RNA-seq data of glioblastoma, prostate cancer, lung cancer, breast cancer, and lymphoma from different regions of the World, we have found that *KANSARL* fusion transcripts were rarely detected in the tumors of individuals from Asia or Africa. In contrast, they exist in 30 - 52% of the tumors from North Americans cancer patients. Analysis of CEPH/Utah Pedigree 1463 has revealed that *KANSARL* is a familially-inherited fusion gene. Further analysis of RNA-seq datasets of the 1000 Genome Project has indicated that *KANSARL* fusion gene is specific to 28.9% of the population of European ancestry origin. In summary, we demonstrated that *KANSARL* is the first cancer predisposition fusion gene associated with genetic backgrounds of European ancestry origin.

## INTRODUCTION

Genetic predisposition to cancer has been well known for several centuries initially through observation of unusual familial clusterings, and later through identification of cancer-prone families that demonstrate Mendelian inheritance of cancer predisposition using different traditional techniques such as comparative genomic hybridization [1–4]. Over 100 cancer predisposition genes (CPGs) have been identified, including *BRCA1* and *BRCA2* in breast cancer, TP53 in Li–Fraumeni syndrome, and APC in familial adenomatous polyposis [1]. All these CPGs are derived from known genes carrying point mutations; however, none of them are derived from gene fusion [1]. Since the genetic factors identified so far only explain a small percentage of familial cancer risks, discovery of novel genetic predisposition factors for cancer is needed [5]. Fusion transcripts, which can be derived from chromosomal rearrangements and RNA processing events such as *cis*- and *trans*-splicing [6], are of particular importance in that their products are able to elicit immune responses in cancers and other diseases and therefore may serve as cancer-specific antigens for cancer vaccine development [7–15], a field that is currently drawing extensive attention for cancer immunotherapy [7, 16–21].

Recent advances in RNA-seq have made it possible to systematically analyze human cancer transcriptomes for the discovery of fusion transcripts [10, 11, 22–25]. In recent years, RNA-seq data have grown exponentially, and around 30,000 novel fusion transcripts and genes have been identified thus far [26, 27]. The key challenge of using RNA-seq to identify fusion transcripts lies in mapping RNA-seq reads quickly and accurately to the reference genome [28]. Although enormous progress has been made and more than 30 software and algorithms have been developed for this purpose, these systems are of low efficiency, sensitivity and accuracy although great efforts have been made to improve [6, 29–32]. For example, Kinsella et al. have developed them a method that allows to ambiguously map RNA-seq reads, and used it to identify *KANSL1*-*ARL17A* (*KANSARL*) fusion transcripts [33]. However, its fusion junction has turned out to be identical to a cDNA clone [34]. Additionally, details about this fusion that of transcript remain to be elusive, including its expression patterns, relationship to somatic and germline mutations, and association with cancer types and genetic backgrounds.

Both *ARL17A* and *KANSL1* genes are located on the reverse strand of the chromosome 17q21.31. *KANSL1* encodes an evolutionarily-conserved nuclear protein, a subunit of MLL1 and NSL1 complexes that is involved in histone H4 acetylation and p53 Lys120 acetylation [35]. *KANSL1* ensures faithful chromosome segregation during mitosis [36]. Two haplotypes of *KANSL1* have been described; the H1 and the inverted H2 forms of 17q21.31 polymorphism. Both contain independently derived, partial duplications of the *KANSL1* gene. These duplications have recently arisen with high frequencies (26% and 19%) in the population of European ancestry origin [37]. Some *KANSL1* mutations have resulted in Koolen-de Vries syndrome (KdVS) (OMIM #610443), characterized by developmental delay, intellectual disability, hypotonia, epilepsy, characteristic facial features, and congenital malformations in multiple organs [38]. *ARL17A* gene encodes a protein of the ARF family that is involved in multiple regulatory pathways relevant to human carcinogenesis [39].

Previously, we have reported that recently gained human spliceosomal introns have a signature of identical 5’ and 3’ splice sites [40]. Based on this finding, we have found that both 5’ exonic sequences (E5) immediately upstream of introns and 3’ intronic sequences near the 3’ splice site (I3) are dynamically conserved among different lineages of eukaryotic introns. The conservation is reminiscent of self-splicing group II introns and of constraints imposed by base pairing between intronic-binding sites (IBSs) and exonic-binding sites (EBSs) [40]. Therefore, we propose that both E5 and I3 sequences constitute splicing codes, which are deciphered by as-yet-to-be characterized splicer proteins/RNAs via base-pairing [40]. Using this splicingcode model, we developed a computational tool for analyzing RNA-seq datasets in order to study gene expression pattern, and to identify novel splicing isoforms and fusion transcripts.

In this study, we have used this software system to analyze RNA-seq data from a variety of cancer types, and identified over 1 million fusion transcripts of unique splice junctions. To verify the reliability and robustness of our approach, we selected *KANSARL* fusion transcripts for systematic validation and characterization. We have found that *KANSARL* fusion transcripts are associated with multiple types of cancer. We have further shown that *KANSARL* represents the first predisposition fusion gene specific to the population of European ancestry origin.

## RESULTS

### Development of a computational high throughput tool, SCIF (SplicingCodes Identify Fusion Transcripts), for identification of fusion transcripts

To develop an improved computational program for identification of fusion transcripts, we first generated a human splicingcode table as described previously [40, 41]. Supplementary Figure 1 shows the flowchart of using the human splicingcode to identify fusion transcripts from human RNA-seq data. After removal of highly-repetitive sequences, poor quality sequences, and duplicated sequences, we reduced our collection of introns from 382,279 to 230,000, the majority of which are unique. To identify fusion transcripts, we selected 20-bp sequences as key length, and screened our 230,000 pairs of 5’ and 3’ exonic keys. To improve the alignment accuracy and speed, we have added an important step to continue sequence alignments, that is, the sequences upstream of the 5’ key and downstream of the 3’ key of the RNA-seq read were further aligned to the corresponding genomic region of 5’ and 3’ exons. Supplementary Figure 2 shows that the 5’ region of an RNA-seq read is identical to the 5’ exonic key from Gene A, and its immediately downstream sequences were aligned to the 3’ exonic key of Gene B. If both remaining regions are identical to the corresponding genomic regions, this read was then blasted against AceView human mRNAs/EST database and the human gene database containing 20-kb upstream sequences of 5’ genes and 20-kb downstream sequences of 3’ genes, to remove sequences from pseudogenes, gene duplications and alternatively-spliced sequences. If blasted results confirmed that the RNA-seq read was originated from two different regions or genes, this read was deemed as a fusion transcript. To minimize potential errors, we randomly generated exon-exon and exon-intron chimeric sequence datasets to check artificial exon-exon and exon-intron chimeric sequences. We analyzed over 20,000 million of 75-101 bp RNA-seq reads, and did not find a single copy of such random chimeric sequences, suggesting that the artifacts generated during the experiments have been reduced to close to zero. Because our system requires that both 5’ and 3’ keys of fusion transcripts are in the splicingcode table and their remaining sequences are identical to the corresponding genomic sequences of the keys, this approach allows us to remove poor-quality and repetitive reads quickly and to have a very fast speed in aligning the entire reads to their genomic targets with much higher accuracy. Consequently, based on the lengths of identical sequences, we found that the maximum of random error to generate a fusion transcript was 1.2 × 10^-24^ and the medium error was 1 × 10^-59^. We have named this program SCIF (SplicingCodes Identify Fusion Transcripts).

### Identification of *KANSARL* fusion transcripts

**Figure 1:**
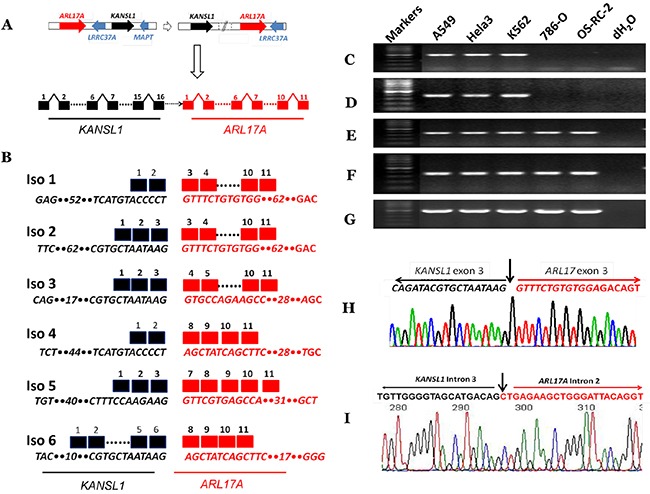
Identification and characterization of *KANSARL* (*KANSL1*-*ARL17A*) fusion transcripts **(A)** A schematic diagram showing steps of genetic rearrangements from normal genomic structures of *ARL17A* → *KANSL1* genes to inverted genomic structures of *KANSL1* → *ARL17A* genes on the chromosomal band 17q21.31. Dashed white horizontal arrow and solid white vertical arrow represent genomic rearrangements and potential fusion gene structures. Solid red and black horizontal arrows indicate *ARL17A* and *KANSL1* genes, respectively. Solid blue arrows represent *LRRC37A* and *MAPT* genes, respectively. The dashed horizontal black arrow indicates undetermined genomic regions. Black and black squares represent *KANSL1* and *ARL17A* exons respectively. **(B)** The schematic diagram shows *KANSARL* fusion transcripts identified so far. Black and red squares represent *KANSL1* and *ARL17A* exons respectively. Dashed lines indicate omitted regions. The numbers above the black and red squares are exon numbers. The numbers within sequences indicate omitted numbers of nucleotides; **(C)** Validation of *KANSARL* isoform 1 in A549, HeLa, K562, 786-O and OS-RC-2 cell lines; **(D)** Validation of *KANSARL* isoform 2 in A549, HeLa, K562, 786-O and OS-RC-2 cell lines; **(E)** Detection of *KANSL1* gene expression in A549, HeLa, K562, 786-O and OS-RC-2 cell lines; **(F)** Detection of *ARL17A* gene expression in A549, HeLa, K562, 786-O and OS-RC-2 cell lines; **(G)** Detection of *GAPDH* gene expression as loading controls in A549, HeLa, K562, 786-O and OS-RC-2; **(H)** Sanger sequencing validation of *KANSARL* isoform 2. The black and red letters represent *KANSL1* exon 3 and ARL17A exon 3 sequences, respectively. And **(I)** Sanger sequencing validation of *KANSARL* genomic breakpoint in the Hela-3 cell line. The black and red letters indicate *KANSL1* and *ARL17A* intronic sequences, respectively. Vertical arrows indicate the fusion junctions. The black and red lines indicate *KANSL1* and *ARL17A* sequences, respectively. All markers are 100 bp DNA markers.

We first used the above approach to analyze 37,208 millions of RNA-seq reads from 39 cancer cell lines (designated as ECD39), majorities of which were downloaded from the ENCODE project [42]. We identified a total of 92,817 fusion transcripts with unique fusion junctions. We then selected *KANSARL* fusion transcripts for further investigation. The existence and abundances of multiple *KANSARL* isoforms in cells ruled out the possibility that *KANSARL* fusion transcripts are *trans*-spliced products. The *ARL17A* and *KANSL1* genes consist of 11 and 16 exons, respectively (Figure 1A). An inverted genomic structure of *KANSL1*→*ARL17A* gene order to generate *KANSARL* fusion transcripts results from putative inversions or duplications of the normal genomic structure, *ARL17A*→ *KANSL1* (Figure 1A). Figure 1B and Supplementary Table 1 show that the six *KANSARL* isoforms 1 to 6 identified in ECD39 datasets. In these six *KANSARL* isoforms, *KANSL1* gene uses three splice junctions of exons 2, 3, and 6, indicating that the 5’ breakpoint occurred at least downstream of the exon 2. *ARL17A* retains exons 3, 4, 7, and 8, indicating that the 3’ breakpoint occurred upstream of the *ARL17A* exon 3 (Figure 1B). It is notable that only *KANSARL* isoform 2 among these six *KANSARL* isoforms was identified previously [33, 34], and the other five isoforms are novel. Supplementary Figure 3 shows that the six *KANSARL* fusion transcripts encode putative peptides with 437, 483, 496, 505, 450, and 637 amino acids, respectively. The majority of these peptide sequences were derived from *KANSL1* gene. Thus, these putative KANSARL fusion peptides are similar to those derived from *KANSL1* truncated mutants and retain only coiled-coil domain, while lost the WDR5 binding region, Zn finger, the domain for KAT8 activity, and PEHE [38].

### Experimental validation of *KANSARL* fusion transcripts and genomic breakpoint

We next aimed to validate *KANSARL* fusion transcripts shown in Figure 1B. To this end, we used A549, HeLa, and K562 cells, which are positive for *KANSARL* fusion transcripts in RNA-seq datasets (Supplementary Table 2), for RT-PCR and sequencing analyses, and cell lines 786-O and OS-RC-2 were used as negative controls. Figure 1C shows that amplification of A549, HeLa, and K562 cDNAs using the primer pair KANSARLISOF1 and KANSARLISOR1 generated PCR fragments with the expected size 379-bp (Supplementary Table 4). Direct Sanger DNA sequencing of the cloned PCR fragments confirmed that they have the correct fusion junction of *KANSARL* fusion isoform 1 (Supplementary Figure 4). Figure 1D shows that PCR using the primer pairs KANSARLF1 and KANSARLR1 (Supplementary Table 4) produced fragments with the expected size 431-bp. Sequencing analysis shows that these fragments contain the expected fusion junction of *KANSARL* isoform 2 (Figure 1H), which was reported previously [33, 34]. Overall, we have shown that both *KANSARL* isoforms 1 and 2 contain the correct fusion junction sequences.

To better understand *KANSARL* fusion events, we performed RT-PCR analysis for *KANSL1* and *ARL17A* expression using primers overlapping breakpoints of *KANSL1* and *ARL17A* genes (Supplementary Table 4) in *KANSARL*-positive (A564, HeLa, and K562) and *KANSARL*-negative (786-O and OS-RC-2) cell lines. Figure 1E&1F show that the expression patterns of *KANSL1* and *ARL17A* genes in A564, HeLa, and K562 are very similar to those in 786-O and OS-RC-2. Figure 1G shows the expression levels of GAPDH gene that were used as loading controls.

To further validate the *KANSARL* genomic breakpoint, we have also performed analysis of whole genome shotgun (WGS) data from *KANSARL*-positive individuals of CEPH/Utah Pedigree 1463 and results indicated that the genomic breakpoints are located at *KANSL1* intron 3 and *ARL17A* intron 2 (Supplementary Figure 5). Then, we have isolated genomic DNA from Hela-3 cells and performed genomic amplification using the primers gKANSL1F1 and gARL17AR1 (Supplementary Table 4). Supplementary Figure 6 shows the PCR product, which was then recovered for direct DNA sequencing (Supplementary Figure 7). DNA analysis and manual inspection have located the breakpoint of *KANSL1* intron 3 and *ARL17A* intron 2 (Figure 1I), and indicated that the PCR product contains the identical genomic breakpoint sequences of the H1 form of the 17q21.31 inversion polymorphism reported previously [43]. Thus, we have validated the *KANSARL* fusion breakpoint at both RNA and genome levels and derived from the 17q21.31 inversion polymorphism reported previously [43].

### Characterization of the expression patterns of *KANSARL* fusion transcripts in ECD39 cancer cell lines

**Figure 2:**
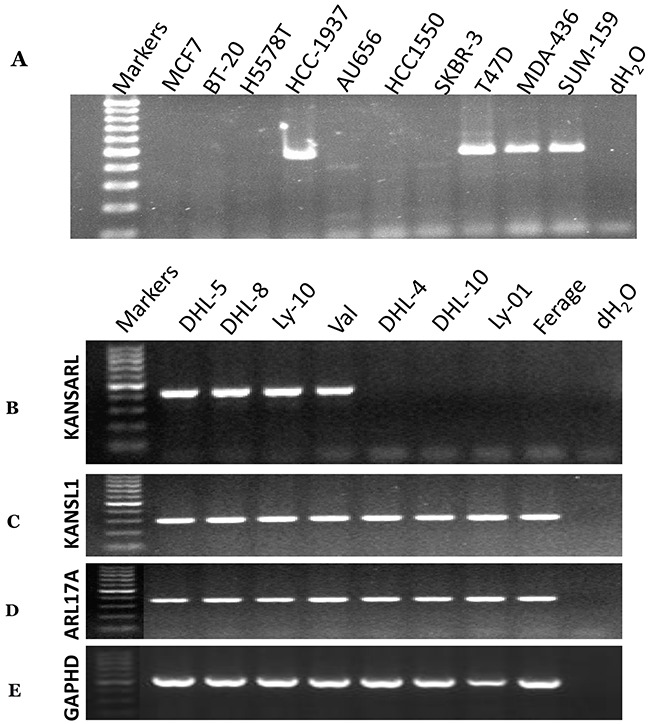
RNA-typing of *KANSARL* fusion transcripts in breast and lymphoma cancer cell lines Detections of *KANSARL* transcripts were evaluated by RT-PCR with the primers shown in Supplementary Table 4. **(A)** breast cancer cell lines; **(B)** lymphoma cell lines. **(C)** Detection of *KANSL1* gene expression; **(D)** Detection of *ARL17A* gene expression; and **(E)** Detection of *GAPHD* gene expression as controls. All markers are 100bp DNA markers;

As shown in Supplementary Table 2, 11 out of 39 (29%) cancer cell lines express *KANSARL* fusion transcripts, suggesting that *KANSARL* is a highly-recurrent fusion gene. To verify this claim, we performed RT-PCR analysis for a pool of uncharacterized breast cancer and lymphoma cell lines. Figure 2A shows that among 10 breast cancer cell lines, HCC-1937, T47D, MAD-436, and SUM-157 cells express *KANSARL* isoform 2. Figure 2B shows that among the eight lymphoma cell lines, DHL-5, DHL-8, OCI-Ly10, and Val express *KANSARL* isoform 2. Figure 2C&2D show that all the eight lymphoma cell lines have at least one copy of *KANSL1* gene and one copy of *ARL17A* gene. Thus, the results show that *KANSARL* fusion transcripts exist in cancer cell lines at high frequencies.

**Figure 3:**
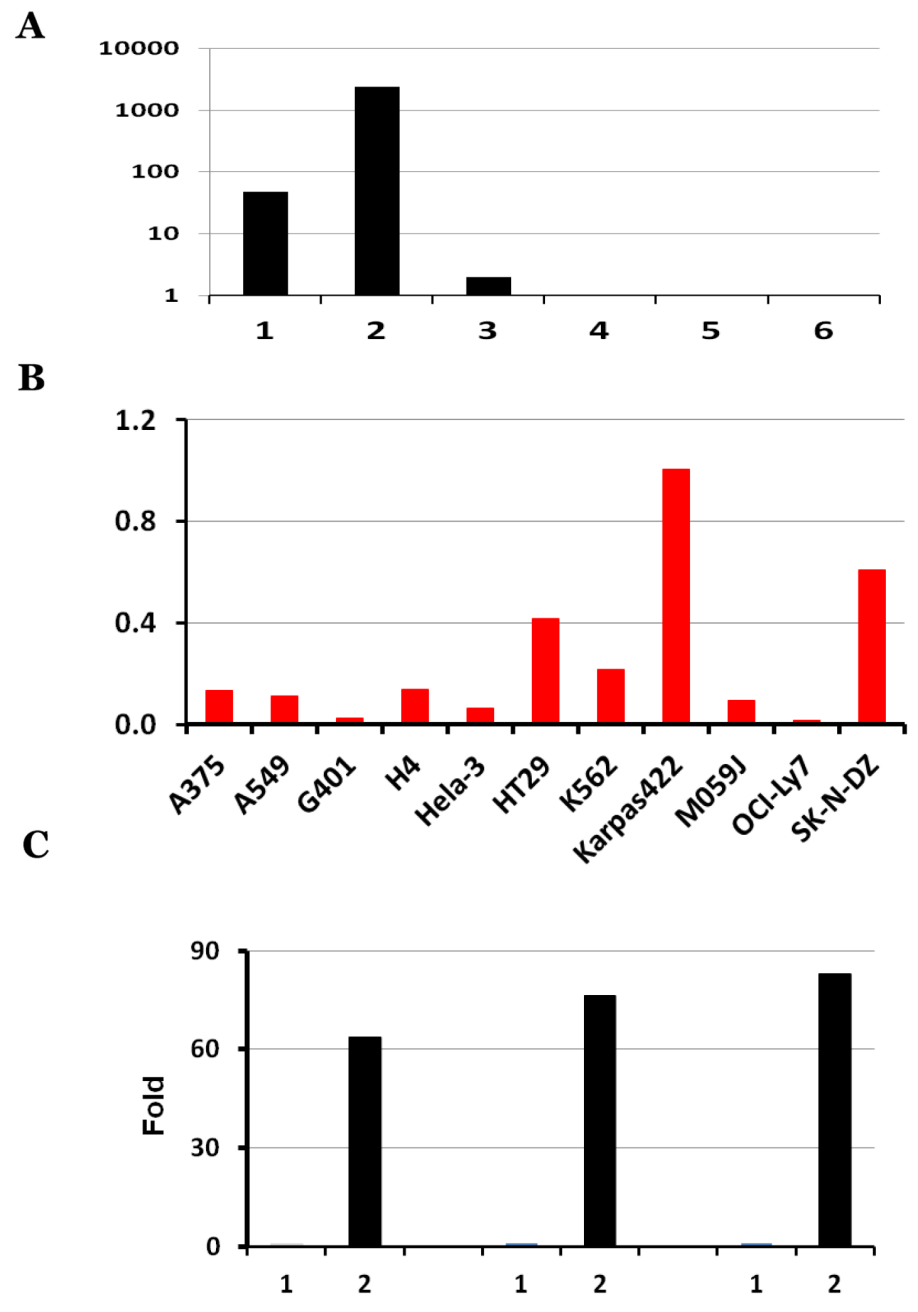
Characterization of *KANSARL* isoform expression in ECD39 cell lines, A549, HeLa and K562 **(A)** Distribution of raw counts of the six *KANSARL* isoforms identified in the ECD39 datasets; **(B)** Normalization of raw counts into NSJMR (Numbers of Splice Junctions per Million) of *KANSARL* fusion transcripts among the *KANSARL*-positive cancer cell lines. Y-axis shows Numbers of Splice Junctions per Million Reads (NSJMR). **(C)** Quantification of *KANSARL* isoform 1 and 2 of A549, HeLa and K562 by real-time PCR with the primers shown in Supplementary Table 4. The raw real-time RT-PCR data of *KANSARL* isoform 1 and 2 of A549, HeLa and K562 are listed in Supplementary Table 3.

To further investigate the differential expression of these six individual *KANSARL* isoforms in cancer cell lines, we analyzed their relative abundance of in ECD39 dataset [42]. Figure 3A and Supplementary Table 1 show the distribution of raw counts of the six *KANSARL* fusion transcripts, among which *KANSARL* isoform 2 is the most abundant, and is 50-fold and 1,216-fold greater than isoforms 1 and 3, respectively. Supplementary Table 2 shows that *KANSARL* fusion transcripts were detected in 11 out of 39 cancer cell lines, including A375, A549, G401, H4, HeLa-3, HT29, K562, Karpas422, M059J, OCI-Ly7, and SK-N-DZ. In contrast, we have not identified a single copy of KANSARL fusion transcript in the rest of 28 cell lines, among which HepG2 has the largest number (over 1,200 million) of RNA-seq reads analyzed so far, suggesting that the depths of RNA-seq reads have no impacts on our analysis outcomes. To normalize for a total number of reads, we adjusted our values relative to the number of splice junctions per million reads (NSJMR). We found that all the 11 cell lines express *KANSARL* isoform 2 (Figure 3B), and different expression levels of the other five isoforms. Karapas-422, A549, H4, HT29, A375, SK-N-SH, and K562 cells are among the cancer cell lines with the highest levels of *KANSARL* fusion transcripts, and are more than 7-fold on average than the other four cell lines.

Quantitative real-time PCR results indicate that *KANSARL* isoform 2 is expressed at 0.36%, 0.28% and 1.28% of the *GAPDH* expression levels in A549, HeLa, and K562, respectively, while *KANSARL* isoform 1 is expressed only at 0.0056%, 0.0037% and 0.015% of the *GAPDH* expression levels in the same cell lines (Supplementary Table 3). Figure 3C shows that *KANSARL* isoform 2 is expressed at 64 to 83 fold higher than *KANSARL* isoform 1. The differential expression patterns between these two *KANSARL* isoforms detected using real-time PCR are generally consistent with that obtained from RNA-seq data analysis (Supplementary Table 1).

### *KANSARL* fusion transcripts are expressed predominantly in cancer patients with European ancestry origin

**Figure 4:**
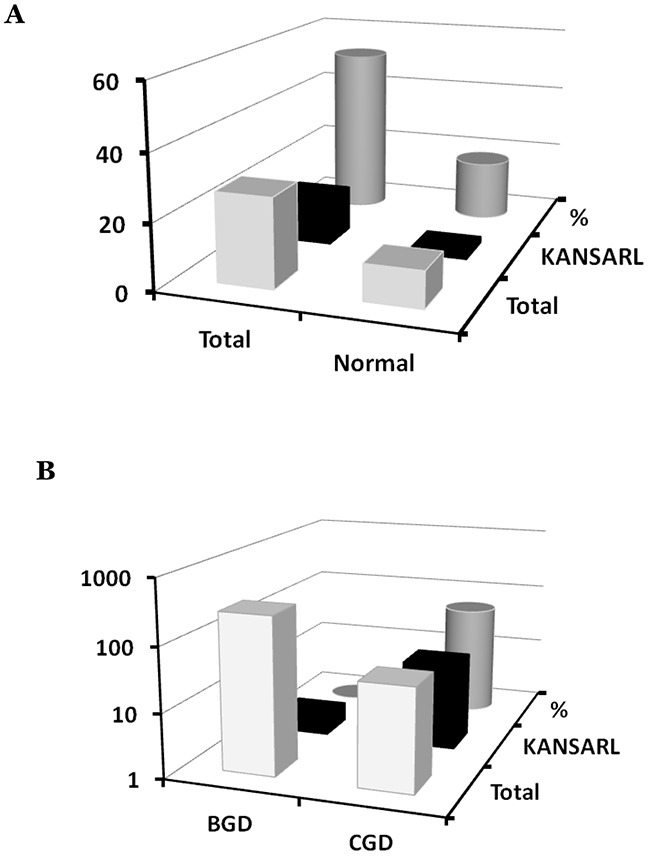
Systematic analyses of *KANSARL* fusion transcripts in glioblastomas **(A)** Analysis of *KANSARL* fusion transcripts in the CGD glioblastoma RNA-seq datasets. “Normal” and “Gliomas” represented brain tissues of non-neoplastic donors and diffuse glioblastomas (GBM) patients, respectively. **(B)** Comparative analysis of *KANSARL* fusion transcripts between the BGD and CGD datasets that consist of 272 glioblastoma patients from Beijing Neurosurgical Institute, Beijing, China, and 27 glioblastoma patients of Columbia University Medical Center, New York, USA, respectively. Comparative analyses of prostate cancer, lung cancer, breast cancer and lymphomas from different geological regions of the World are shown in Supporting Materials.

Since Supplementary Table 2 shows that *KANSARL* fusion transcripts are expressed in brain cancer cell lines including H4, MJ059 and SK-N-DZ, we were prompted to identify and characterize *KANSARL* fusion transcripts in glioblastomas. To this end, we analyzed the glioblastoma RNA-seq dataset from Columbia University Medical Center, New York (designated as CGD), which has a total of 94 samples from 27 glioblastoma patients and 17 non-neoplastic brain tissues [44]. Figure 4A and Supplementary Table 5 show that 14 of the 27 (51.9%) GBM patients express *KANSARL* fusion transcripts. In contrast, *KANSARL* fusion transcripts were detected only in 2 out of 17 (or 11.8%) non-neoplastic brain tissues. The difference in the expression frequencies of *KANSARL* fusion transcripts between glioblastomas and non-neoplastic persons is statistically significant (Supplementary Table 5, Fisher's exact test, p<0.01; Figure 4A), indicating that *KANSARL* fusion transcripts are preferentially associated with diffuse glioblastomas.

To further investigate *KANSARL* fusion transcripts in glioblastomas, we performed a comparative expression analysis of the glioblastomas dataset deposited by Beijing Neurosurgical Institute, Beijing, China (designated as BGD), which has 272 gliomas of different clinic prognosis stages [45]. Surprisingly, only two out of the 272 BGD glioblastoma samples (less than 1%) are *KANSARL* positive (Figure 4B). Thus, *KANSARL* positivity in BGD glioblastoma is 70 fold lower than that in the CGD dataset. Supplementary Table 6 shows that the difference between CGD and BGD is statistically significant (Fisher's exact test, p<0.001).

The dramatic difference in the expression frequencies of *KANSARL* fusion between the CGD and BGD datasets has raised the possibility that *KANSARL* fusion transcripts are associated with cancer patients of European ancestry origin. To confirm this claim, we performed comparative analyses of RNA-seq datasets in more types of cancers, including prostate cancer, breast cancer, lung cancer and lymphoma from different geological regions of the World (see detailed analysis in Supporting Data). Our results show that *KANSARL* fusion transcripts were rarely found in the tumor samples from Asia and Africa, but were detected in 30~52% of cancer samples from patients in North America. Therefore, we conclude that *KANSARL* fusion transcripts are preferentially associated with tumor samples from patients with European ancestry origin.

### *KANSARL* fusion transcripts are associated with prostate cancer biomarker *TMPRSS2*-*ERG* fusion transcripts in prostate cancer

**Figure 5:**
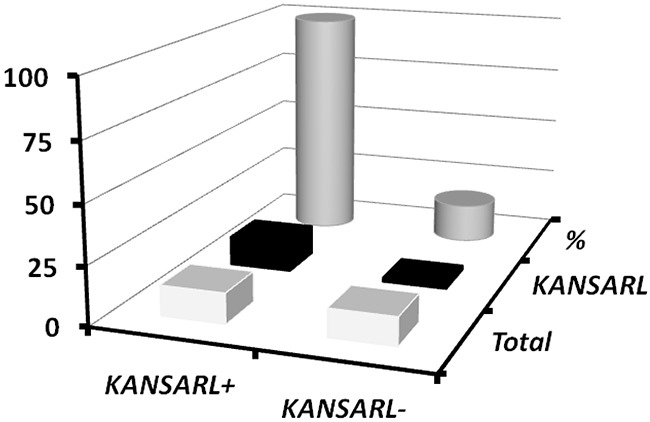
Relationship between *KANSARL* fusion transcripts and somatic *TMPRSS2-ERG* fusion transcripts in VPD prostate tumors The dataset VPD has 25 prostate patient samples from Vancouver Prostate Centre [46]. *KANSARL*^+^ and *KANSARL*^-^ represented *KANSARL*-positive and *KANSARL*-negative tumor samples, respectively. *TMPRSS2*-*ERG* fusion gene is the predominant molecular subtype of prostate cancer.

In supporting data, analysis of 25 high-risk prostate tumors from Vancouver Prostate Center, Vancouver, Canada (VPD) [46] has shown that 13 of them (52%) have *KANSARL* fusion transcripts. We have also found that *KANSARL* and *TMPRSS2-ERG* are the two most highly-expressed fusion genes in the VPD prostate cancer dataset. These observations suggest a possible relationship between *KANSARL* fusion transcripts and somatic *TMPRSS2*-*ERG* fusion transcripts. We have further shown that *TMPRSS2*-*ERG* fusion transcripts are expressed in 15 out of 25 prostate tumors, consistent with the notion that *TMPRSS2*-*ERG* fusion transcripts are prostate cancer biomarkers [46]. Figure 5 and Supplementary Table 7 show that 13 out of 15 *TMPRSS2*-*ERG*-positive prostate tumors are *KANSARL*-positive, and 100% *KANSARL*-positive prostate tumors express somatic *TMPRSS2*-*ERG* fusion transcripts. In contrast, only two *TMPRSS2*-*ERG*-positive prostate tumors were detected in 12 *KANSARL*-negative prostate tumors. The difference of *TMPRSS2*-*ERG* fusion transcripts between *KANSARL*-positive and *KANSARL*-negative tumors is significant (Supplementary Table 7, Fisher's exact test, p<0.001), indicating that *KANSARL* fusion transcripts are associated with prostate cancer biomarker *TMPRSS2*-*ERG* fusion transcripts in the VPD tumor patients.

Furthermore, Supplementary Table 8 shows that the prostate and breast cancers with *KANSARL* fusion transcripts have increased the numbers of recurrent read-through (epigenetic) fusion transcripts than those without *KANSARL* fusion transcripts, suggesting that *KANSARL* fusion transcripts may be involved in epigenetic alternations in early stages of cancer development.

### *KANSARL* fusion gene is familially inherited and may be ubiquitously expressed in *KANSARL*-bearing individuals

**Figure 6:**
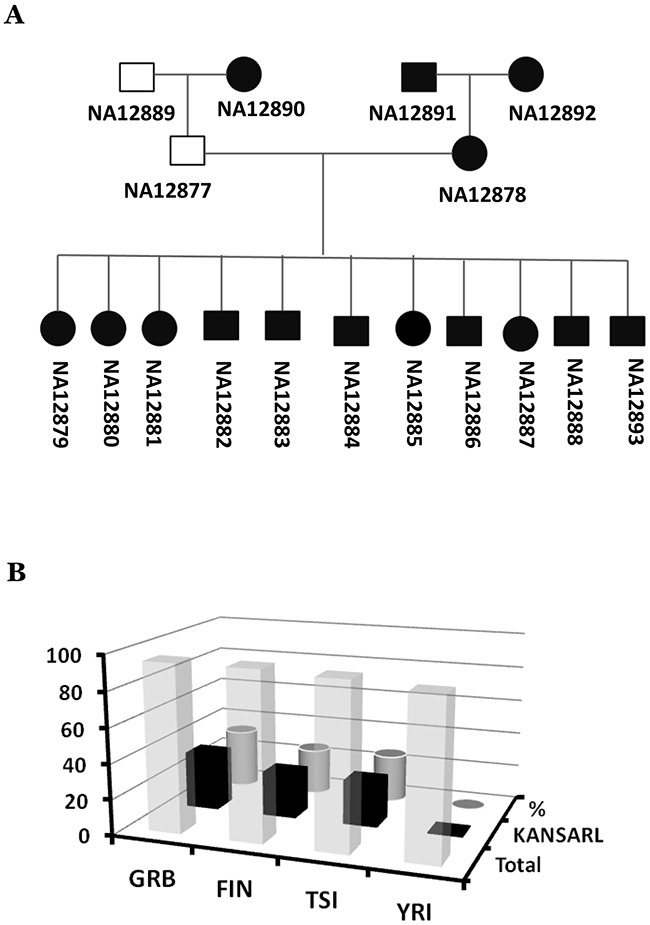
Inheritance and distribution of *KANSARL* fusion transcripts in the population of European ancestry origin **(A)** Diagrams of correct *KANSARL* inheritance in the CEPH/Utah Pedigree 1463, which includes four grandparents, two parents, and eleven children. Black and white squares represent *KANSARL*-positive and *KANSARL*-negative males while black and white squares indicate *KANSARL*-positive and *KANSARL*-negative females. The black lines represent relationships among the family members. The diagram is drawn based on RNAs-seq and WGS data. **(B)** Analysis of *KANSARL* fusion transcripts in the RNA-seq data of the lymphoblastoid cell lines of the 1000 Genome Project [48]. The diagram shows frequencies of *KANSARL* fusion transcripts in some populations of European and African ancestries. GBR is British from England and Scotland; FIN indicates Finnish in Finland; TSI represents Toscani in Italia, and YRI is Yoruba in Ibadan, Nigeria.

The presence of *KANSARL* fusion transcripts in normal and adjacent tissues in CGD and VPD raised the possibility that *KANSARL* fusion transcripts are derived from a germline-inherited fusion gene. To investigate this possibility, we performed RNA-seq data analysis for the lymphoblastoid cell lines derived from the families in the CEU population (CEPH/Utah Pedigree 1463, Utah residents with ancestry from northern and western Europe), which includes a three-generation family with 17 individuals [47]. Supplementary Table 9 and Supplementary Figure 8 show that *KANSARL* fusion transcripts were detected in 15 of 17 family members, as indicated by black squares and circles in Supplementary Figure 8, except son (NA12885), which is deviated from the first Mendel law. A reasonable explanation is that the grandfather sample (NA12889) might have been mixed with the son sample (NA12885). To prove this possibility, we performed analyses of the WSG data (PRJEB3381) and RNA-seq from 1000 Genome Project, and shown that both WSG and RNA-seq of grandfather sample (NA12889) are *KANSARL*-negative while WGS of the son (NA12885) is *KANSARL*-positive (Supplementary Table 10). Supplementary Table 10 shows the genomic breakpoint 1 and 2 of the *KANSARL* fusion gene (Supplementary Figure 5 and 9) identified by analysis of WGS data among some members of CEPH/Utah Pedigree 1463. Therefore, Both WGS and RNA-seq data support that the father (NA12877) and the mother (NA12878) have the genotypes of *KANSARL*^-^/*KANSARL*^-^ and *KANSARL*^+^/*KANSARL*^+^ respectively and all their offsprings are the genotype *KANSARL*^+^/*KANSARL*^-^ (Figure 6A).

As shown above, *KANSARL* is a familially-inherited fusion gene. A critical question was then whether *KANSARL* fusion transcripts exist in general populations. To answer this question, we analyzed RNA-seq data of the lymphoblastoid cell lines of the 1000 Genome Project [48]. Supplementary Table 11 shows that no single copy of *KANSARL* fusion transcripts has been detected in the Nigeria YRI (Yoruba in Ibadan) population. In contrast, Figure 6B and Supplementary Table 11 show that *KANSARL* fusion transcripts have been found in 33.7% GBR (British from England and Scotland), 26.3% FIN (Finnish in Finland) and 26.9% TSI (Toscani in Italia) populations, respectively. The differences of *KANSARL* frequencies among the GBR, FIN, and TSI population are not statistically significant (data not shown), suggesting that these differences may be caused by sampling errors. However, their differences with that of Nigeria YRI are statistically significant (Supplementary Table 11, Fisher's exact test, p<0.001), confirming our claim that *KANSARL* fusion transcripts are specific to the population of European ancestry origin.

As Supplementary Table 2 shows, *KANSARL* fusion transcripts are expressed in many tissues and organs in *KANSARL*-bearing individuals. To systematically understand the patterns of *KANSARL* gene expression in human tissues, we analyzed RNA-seq datasets from Science for Life Laboratory, Sweden (designated as SSTD), which are originated from 32 different tissue samples of 127 healthy individuals [49]. Supplementary Table 12 shows that *KANSARL* fusion transcripts are detected in 28 of 32 tissues analyzed, except bone marrow, kidney, stomach and smooth muscle. These data suggest that *KANSARL* fusion transcripts are ubiquitously expressed in the tissues and organs of *KANSARL*-bearing individuals.

## DISCUSSION

In this study, we have developed and used our high throughput computational system SCIF to systematically analyze *KANSARL* fusion transcripts in a variety of cancer and normal samples with high capacity, high sensitivity, high accuracy and much lower computation time consumption. Our results demonstrate that *KANSARL* fusion transcripts are derived from a cancer predisposition fusion gene specific to the population of European ancestry origin.

### Technical improvements for profiling fusion transcripts from RNA-seq datasets

Chimeric RNAs are well known to be generated from chromosomal rearrangements, including translocations, deletions, and inversions. Recently, *trans*-splicing and *cis*-splicing of read-through pre-mRNAs between adjacent genes have been shown as emerging mechanisms for their generation [6]. Intergenically spliced fusion transcripts, which could be present at low levels in a given cancer type, represent a new repertoire of cancer-specific biomarkers for early cancer detection, and also have potential applications in cancer diagnosis, prognosis, and therapeutic design [6, 28].

The major obstacles to analyze RNA-seq data--“Big Data” are to map reads to the genome with high efficiency and accuracy. As shown in Supplementary Figure 1, to improve accuracy and efficiency, first, SCIF has directly mapped raw sequences of RNA-seq reads to the human genomes instead of clustering. Secondly, SCIF has directly extended their flanking sequence alignments after partial sequences of a read were mapped to two different genes, as shown in Supplementary Figure 2. A desktop computer with a core of i5 and 8 GB memory can process 25~120 millions of RNA-seq reads per hour since validation times used by BLAST constitute up to 80% of the computation costs and are highly variable. The SCIF software can detect a single copy of a given fusion transcript to over 10,000 fusion transcripts per sample.

In our practice, to generate consistent *KANSARL* data, we have set the minimum 20 million RNA-seq reads for *KANSARL* analysis. These quality controls have greatly increased data reproducibility and minimized misalignment errors to false positivity less than 1%. For example, the CGD 27 glioblastoma dataset includes 39 CE samples and 36 NE sequencing samples that were effectively constituted as multiple duplicate experiments in the context of *KANSARL* fusion transcripts [44]. All *KANSARL*-positive samples were detected in the corresponding CE and NE samples and the duplicate samples, and all *KANSARL*-negative samples are also reproducible in analysis results. That is, analysis results of 100% of both *KANSARL*-positive and *KANSARL*-negative samples can be reproducible, indicating that our approach is reliable.

Moreover, analysis of the VPD prostate cancer RNA-seq dataset using SCIF has identified 16 *TMPRSS2-ERG* fusion transcripts (rearrangements), 11 of which are novel (manuscript in preparation). Thus, our technology overcomes the major challenges and limitations in previous techniques, and provides a powerful tool for detection of low-level fusion transcripts that can serve as biomarkers for early cancer diagnosis [6].

### *KANSARL* fusion gene is associated with polymorphism of the chromosomal band 17q21.31

The identical fusion junction *of KANSARL* fusion transcripts and genomic breakpoint of the *KANSARL* fusion gene (Figure 1H and Supplementary Figure 7) with those from the H1 form of 17q21.31 inversion polymorphism suggest that *KANSARL* fusion transcripts are derived from the H1 form of 17q21.31 inversion polymorphism, which counts for 26% population of European ancestry origin [43]. Since we have excluded the data from Utah residents with ancestry from northern and Western Europe like those of CEPH/Utah Pedigree 1463, average 28.9% KANSARL fusion transcripts in population of European ancestry origin are much higher than 26% of the H1 form [43]. One of possibilities is that complexities of duplications make it difficult to perform accurate analysis of WGS data and result in lower numbers of the H1 form. The second possibility is that additional genomic breakpoints exist in the chromosomal band 17q21.31. The presence of the putative genomic breakpoint 2 of *KANSARL* fusion gene in the CEPH/Utah Pedigree 1463 supports this possibility (Supplementary Figure 9 and Supplementary Table 10). However, the additional *KANSARL* genomic breakpoint will be required for further confirmation. This uncertainty of the putative *KANSARL* fusion genomic breakpoints suggests that RNA-typing of *KANSARL* fusion transcripts will provide more accurate data than DNA-typing of the *KANSARL* genomic breakpoints.

### *KANSARL* as the first cancer predisposition fusion gene that may play crucial roles in tumorigenesis

We have provided solid evidence, for the first time, supporting that the *KANSARL* fusion transcripts are associated with multiple types of cancer, including glioblastomas, prostate, breast cancer, lung cancer and lymphomas. *KANSARL* fusion transcripts have been detected in healthy individuals of European ancestry origin and are familially-inherited. These results support our claim that *KANSARL* is a cancer predisposition fusion gene specific to populations of European ancestry origin.

In all analyzed datasets, we have detected *KANSARL* fusion transcripts in all samples with *KANSARL* fusion gene, and the isoforms 1 and 2 have significantly higher expression levels in cancer patients compared to healthy individuals in general. However, isoforms 3-6 are expressed at very low levels and their differences cannot be determined.

All the six putative fusion peptides encoded by *KANSARL* fusion transcripts lack some functional domains, and therefore cancer patients expressing *KANSARL* fusion transcripts would display reduced activities of the histone acetyltransferase KAT8 and p53 [50–52]. It is quite possible that the reduction of KAT8 and p53 activities results in hypermutations in certain chromosomal regions of cancer cells and/or epigenetic changes that generate new read-through fusion transcripts, as shown in Supplementary Table 8. Since *KANSARL*-bearing cancer patients may be not sensitive to histone deacetylase inhibitors (HDACis) due to lower histone acetylation, *KANSARL*-negative patients may be respond well to treatments of HDACis.

In summary, our results demonstrate that *KANSARL* fusion gene exists in many types of cancer and is the first novel germline fusion gene specific to the genetic backgrounds of European ancestry origin. Our research provides an example to use our SCIF system for discovery of novel fusion transcripts that may play underappreciated but crucial roles in tumorigenesis and have promising therapeutic applications by serving as cancer biomarkers, drug targets, or specific epitopes for cancer vaccine development.

## MATERIALS AND METHODS

### Isolation of total RNAs from cell lines

Medium was removed from100-mm culture dishes and 1 ml of Trizol reagent (Invitrogen, CA) was added directly in each 100-mm culture dish. The cells were lysed by vortex vigorously for 15 seconds and the mixes were incubated at room temperature for 2-3 min. The samples were then centrifuged at 4,000 g for 15 min to separate the mixtures into a lower red, phenol-chloroform phase and a colorless upper aqueous phase. RNA in the aqueous phase was precipitated by mixing with 0.5 volumes of isopropyl alcohol for 10 min, and then centrifuging at 12,000 g for 10 min at room temperature. The RNA pellet was washed twice with 1 ml of 75% ethanol, air-dried, and then dissolved in 40-80 μl of RNase-free H_2_O. The organic phase was saved for isolation of DNA or protein.

### cDNA synthesis

The first-strand cDNA synthesis was carried out using oligo(T)_15_ and/or random hexamers using TaqMan Reverse Transcription Reagents (Applied Biosystems Inc., Foster City, CA, USA) as suggested by the manufacturer. In brief, to prepare 2× RT master mix, we pooled 10 μl of reaction mixes containing final concentrations of 1X RT Buffer, 1.75 mM MgCl_2_,, 2 mM dNTP mix (0.5 mM each), 5 mM DTT, 1X random primers, 1.0 U /μl RNase inhibitor and 5.0 U/μl MultiScribe reverse transcriptase. 10 μl of total RNAs (2 μg) were mixed well with 10 μl of 2X master mixes. The reaction mixes were then placed in a thermal cycler for 10 min at 25°C, 120 min at 37°C, and then for 5 min at 95°C. The resulted cDNAs were diluted with 80 μl H_2_O.

### End-point PCR amplification

To identify novel human fusion transcripts, specific primers were designed to match the 5’ and 3’ ends of a given fusion transcript, using a primer-designing software [53]. 5 μl of the cDNAs generated above were used as template for end-point PCR, which was carried out using HiFi Taq polymerase (Invitrogen, Carlsbad, CA, USA) for 35 cycles of 94°C for 15”, 60-68°C for 15” and 68°C for 2-5 min. The PCR products of *KANSARL* isoforms 1 and 2 were separated on 2% agarose gels, recovered and cloned into TOPO pCR2.1 vector as suggested by manufacturer. After transformation and incubation at 37oC overnight, the plasmids with inserts were isolated and sequenced. The sequences were then verified by blast and manual inspection. For genomic fusion validation, genomic DNA from HeLa-3 cells was used as template for end-point PCR using the Phusion PCR kit (NEB), with an annealing temperature at 60°C.

### Quantitative real-time PCR

To quantify expression levels of different *KANSARL* isoforms, isoform-specific primers were designed using a primer-designing software [53]. 5 μl of the cDNAs generated above were used as template for real-time PCR, which was carried out using SYBR Green PCR Master Mix (Roche) on a LightCycler 480II system (Roche) as the manufacturer suggested. For each reaction, 5 μl of 480 SYBR Green I Master Mix (2X), 2 μl of primers (10X) and 3 μl of H_2_O were pooled into a tube and mixed carefully by pipetting up and down. 15 μl of the PCR mix were pipetted into each well of the LightCycler® 480 Multiwell Plate, and then mixed with 5 μl of cDNAs. The plate was sealed, centrifuged at 1,500 g for 2 min, and then transferred into the plate holder. The PCR was performed for 45 amplification cycles.

### Further information

See Supplementary Materials and Other Supporting Data

## SUPPLEMENTARY MATERIALS FIGURES AND TABLES


