# Supplementary material for: Identification of KANSARL as the first cancer predisposition fusion gene specific to the population of European ancestry origin
Source: Oncotarget. 2017 Mar 24;8(31):50594–607. doi: 10.18632/oncotarget.16385 (PMC5584173; doi:10.18632/oncotarget.16385)
Supplement: Supplementary file 1 [file oncotarget-08-50594-s001.pdf]

Identification of *KANSARL* as the first cancer predisposition fusion gene specific to the population of european ancestry origin

SUPPLEMENTARY MATERIALS AND OTHER SUPPORTING DATA

BRIEF DESCRIPTION OF THE DATASETS USED IN THIS PROJECT

1. **Cancer cell lines (ECD39):** Datasets of the cancer cell lines A172, A375, A431, A549, Caki2, CUTLL1, Daoy, G401, H4, H460, HCC1599, HCT116, Hela-3, HepG2, HT1080, HT29, K562, Karpas422, KATOIII, LHCN-M2, LIM1899, LIM2405, M059J, MCF7, MDA-MB-231, MG63, OCI-Ly7, PC3, REC1, RPMI-7951, SJCRH30, SJSA1, SK-Mel-5, SK-N-DZ, SK-N-SH, SUN16, U251, U2OS and U87MG were used in this project. All of them except REC1, LIM1899, LIM2405, SUN16, U251 and U2OS were downloaded from Encode project (https://www.encodeproject.org/). REC1, KATOIII, LIM1899, LIM2405, SUN16, U251 and U2OS were downloaded from NCBI (Accession: PRJNA176455 and PRJEB3372).

2. **RNA-seq data of CEPH/UTAH family 1463**: CEPH/UTAH family 1463 (Utah residents with ancestry from northern and western Europe from the CEPH collection) has 17 individuals of three generations. RNAs were isolated from Epstein-Barr-virus-transformed peripheral blood B lymphocytes (catalog no. XC01463). Each individuals has obtain 30 million 75 bp paired-end reads (Accession: GSE56961) [1].

3. **WGS data of CEPH/UTAH family 1463**: CEPH/UTAH family 1463 has 17 individuals of three generations. We have selected and downloaded The Platinum genomes (Accession: PRJEB3381) provided by www.platinumgenomes.org to perform KANSARL genomic breakpoints. In The Platinum genomes, Whole genome sequencing (WGS) data of CEPH/UTAH family 1463 have 143-174Gbp paired –end sequences per individuals. We have analyzed the KANSARL genomic breakpoints of the grandparents, parents and partial offspring, which had recently been restricted to access their data and are deposited to dbGap.

4. **The glioblastoma RNA-seq dataset from Columbia University Medical Center (CGD)**: The glioblastoma RNA-seq dataset from Columbia University Medical Center has 27 glioblastoma patients and 11 non-neoplastic brain tissues (unique sample ID) [2]. RNA-seq sequencing was performed on the tissue contained within the contrast-enhancing (CE) core of tumors and tissue from the nonenhancing (NE) margins of tumors per each of 27 glioblastoma patients. Each sample has 1.6 Gbp to 4.2Gbp single sequences (Accession: PRJNA347513).

5. **The glioblastomas RNA-seq dataset from Beijing Neurosurgical Institute (BGD)**: The BGD glioblastomas samples are from Beijing Neurosurgical Institute, Beijing, China and have 272 gliomas of different clinic prognosis stages, which are the largest dataset analyzed in this report [3]. The BGD RNA-seq sequence numbers range from 3.0Gbp to 14.7 Gbp paired-end sequences (Accession: PRJNA212047).

6. **The prostate cancer RNA-seq dataset from Vancouver Prostate Center (VPD)**: The prostate cancer RNA-seq dataset from Vancouver Prostate Center (VPD) is one of the most well-characterized RNA-seq in the numbers of duplications and the large numbers of RNA-seq reads per sample. The VPD contains 25 high-risk primary prostate tumors and five matched adjacent benign prostate tissues [4]. The VPD RNA-seq dataset has performed 85 RNA-seq experiments and generated 2.38 Tb RNA sequences (Accession: PRJEB6530)..

7. **The prostate cancer RNA-seq dataset from Shanghai Changhai Hospital, Shanghai, China (BPD)**: The BPD prostate cancer RNA-seq dataset contains 14 primary prostate cancers and their paired normal counterparts from the Chinese population [5]. The RNA-seq sequences range from 5.6Gbp to 6.9 Gbp (Accession: PRJEB2449).

8. **The lung cancer RNA-seq dataset (designated as SKLCD) from Genomic Medicine Institute, Seoul, Korea (SKLCD)**: The SKLCD dataset contains a normal sample, five more frozen primary lung adenocarcinoma tissues and 200 lung adenocarcinomas in Koreans [6, 7]. We have downloaded and performed analysis of 168 lung cancer samples, whose sequence sizes range from 2.2 Gbp to 22.3 Gbp (Accession: PRJEB2784).

9. **The lung cancer RNA-seq dataset from Michigan Center for Translational Pathology, Michigan USA (MULCD)**: The MULCD lung cancer dataset is part of the dbGaP study “The Landscape of Antisense Gene Expression in Human Cancers” and contains 20 lung tissue samples [8]. The MUCLD dataset contains 11.6 Gbp to 17.7 Gbp paired-end sequences (Accession: PRJNA287193).

10. **The breast cancer dataset from HudsonAlpha Institute for Biotechnology, Alabama USA (HIBCD)**: The HIBCD breast cancer samples are much more complex and consisted of 28 breast cancer cell lines, 42 ER+ breast cancer primary tumors, 30 uninvolved breast tissues adjacent to ER+ primary tumors, 42 triple negative breast cancer (TNBC) primary tumors, 21 uninvolved breast tissues adjacent to TNBC primary tumors and 5 normal breast tissues [9]. The HIBCD RNA-seq sequence sizes range from 2.8 Gbp to 16.2 Gbp. This HIBCD breast

cancer RNA-seq dataset has short 50bp paired-end RNA-seq reads Accession: PRJNA251383). We have noticed that the sequence reads are relatively poor and may result in fewer numbers of KANSARL-positive samples.

11. **The breast cancer RNA-seq dataset from South Korean (SKBCP)**: The SKBCP breast cancer dataset has 22 HRM (high-risk for distant metastasis) breast cancer and 56 LRM (low-risk for distant metastasis) breast cancer patients. It is a part of the study "Integrative systematic analyses of mutational and transcriptional profiles reveal driver mutations escalating the risk of distant metastasis in Korean breast cancers" [10]. The SKBCP sequence sizes range from 4.1Gbp to 27.7 Gbp paired-end sequences (Accession: PRJEB9083).

12. **The Uganda lymphomas dataset from Columbia University College of Physicians and Surgeons, New York, USA (designated as ULD)**: The Uqanda lymphomas contain 20 Endemic Burkitt lymphoma samples, all of which have been collected from the Department of Human Pathology of the Lacor Hospital (Uganda, Africa) [11]. The ULD RNA-seq sequence sizes range from 3.8Gbp to 9.4Gbp (Accession: PRJNA292327).

13. **The Burkitt lymphoma dataset from NCI Metabolism Branch Center for Cancer Research, Bethesda, Maryland, USA (designated as NLD)**: The NLD lymphomas contain 28 sporadic form Burkitt Lymphoma (BL) patient biopsy samples and 13 BL cell lines [12]. The RNA-seq sequence sizes range from 4.1Gbp to 27.0 Gbp paired-end sequences (Accession: SRP009316).

14. **The T-cell lymphoma dataset from Yale School of Medicine, New Haven, Connecticut, USA (designated as YLD)**: The YLD T-cell lymphoma is a part of the study "Genomic landscape of cutaneous T cell lymphoma" and has 13 cutaneous T cell lymphoma [13]. The YLD RNA-seq sequence sizes range from 3.7 Gbp to 25.1 Gbp paired-end sequences (Accession: PRJNA285408).

15. **The diffuse large B-cell lymphoma data from BC Cancer Agency, Vancouver, Canada (designated as BLD)**: The BLD lymphoma RNA-seq dataset is a part of A dbGAP study (study accession: PRJNA172563) [14]. Since RNA-seq read lengths and sequence data sizes are highly variable among different samples, we have selected 23 RNA-seq data of diffuse large B-cell lymphoma, whose RNA-seq sequence data sizes are greater than 2.5 Gbp and their RNA-seq read lengths are at least 50bp longer.

16. **RNA-seq data of 465 lymphoblastoid cell lines from the 1000 Genomes:** Unlike the other RNA-seq data, These RNA-seq datasets from 465 lymphoblastoid cell lines are are from healthy individuals and parts of the 1000 Genomes Project. They have identified 464 different individuals. We have used RNA-seq from GBR (British from England and Scotland), FIN (Finnish in Finland), Nigeria YRI (Yoruba in Ibadan) and TSI (Toscani in Italia) populations. The RNA-seq data from CEU (Utah residents with ancestry from northern and western Europe from the CEPH collection) populations were not used in this study since they come from multiple families and lack diversity and randomness. All of data are 76bp paired end reads and have total 2.54 Tbp data (Accession: PRJEB3366).

17. **The RNA-seq data from HPA normal tissues from Science for Life Laboratory, Sweden (designated as SSTD):** The RNA-seq data were originated from tissue samples of 127 healthy individuals representing 32 different tissues. They have 100bp paired end sequence reads and have total of 500 Gbp data (Accession: PRJEB4337).

OTHER SUPPORTING DATA

Comparative analyses of KANSARL fusion transcripts from prostate, lung, breast cancer and lymphoma

Comparative analysis of prostate cancer RNA-seq data from Canada and China

The dramatic differences of *KANSARL* fusion transcripts between the CGD and BGD have raised the possibility that *KANSARL* fusion transcripts are associated with the cancer patients of European ancestry origins, but absent in cancer patients of Asian ancestry. To confirm this, we have systematically performed comparative analyses of RNA-seq datasets of prostate cancer, breast cancer, lung cancer and lymphomas from different regions around the world.

Prostate cancer is the most common nonskin cancer and the second leading cause of cancer-related death in men in the United States. We have performed analysis of the prostate cancer dataset from Vancouver Prostate Centre (designated as VPD), which contain 25 high-risk primary prostate tumors and five matched adjacent benign prostates tissues [4], and BGI prostate cancer dataset (BPD), which contains 14 pairs of prostate cancer and normal samples [5]. We have detected *KANSARL* fusion transcripts in 13 (52%) out of the 25 VPD prostate samples (Supporting Figure 1) and 4 out of 5 adjacent benign prostate tissues. *KANSARL* isoform 1, 2, and 3 have been detected in the VPD samples and shared very similar patterns to those observed in ECD39. In contrast, we have found no single copy of *KANSARL* fusion transcript in the BPD prostate tumors and their matched normal samples (Supporting Figure 1). Supporting Table 1 shows that the difference between VPD and BPD was statistically significant ($z = 3.118$; $p < 0.05$).

Supporting Table 1: Comparison of number differences of *KANSARL*+ samples between VPD and BPD samples

Types	# of Samples	# of *KANSARL+*	% of *KANSARL+*	Z Scores	Probabilities(p)
**VPD**	25	13	52	3.118	0.002
**BPD**	14	0	0		

Comparative analysis of lung cancer RNA-seq data from USA and South Korea

Lung cancer is the leading cause of cancer deaths in the World, especially in Asia. To investigate the expression of *KANSARL* fusion transcripts, we have analyzed the Korean Lung Cancer RNA-seq dataset (designated as SKLCD), which has 168 lung cancer samples [6] and Michigan of University Lung Cancer Dataset (designated as MULCD), which contains 20 lung tissue samples [8]. We have found that eight (40%) out of 20 MULCD samples have *KANSARL* fusion transcripts (Supporting Figure 2). Even though SKLCD data are more than five folds larger than the MULCD ones, no single copy of *KANSARL* fusion transcripts has been detected in 168 SKLCD samples (Supporting Figure 2). Supporting Table 2 shows that the difference

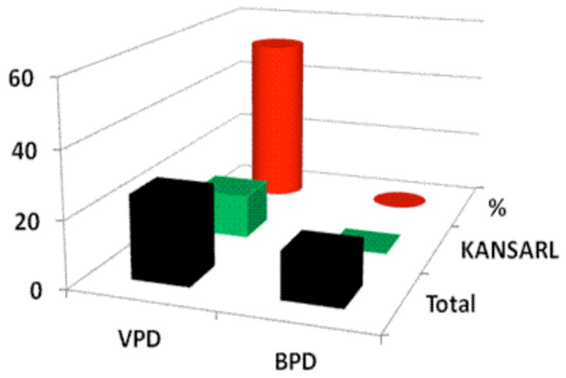

Supporting Figure 1: Comparative analysis of *KANSARL* fusion transcripts between the VPD and BPD datasets. VPD and BPD are 25 prostate patient samples from Vancouver Prostate Centre and 14 prostate tumor samples from Beijing Genome Institute (BGI), respectively. Black and green squares represent total numbers of samples and the numbers of *KANSARL*-positive samples. Red cylinders indicate the percentages *of KANSARL*-positive samples.

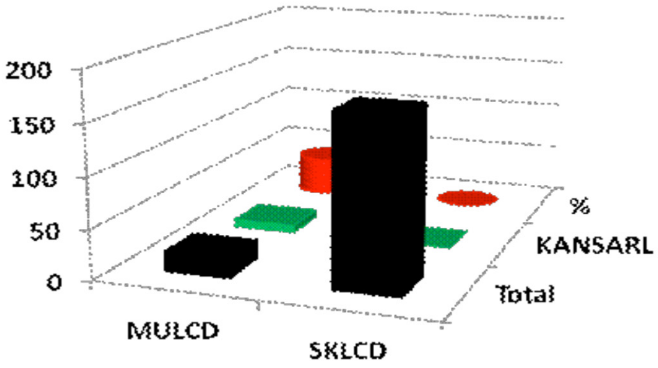

Supporting Figure 2: Comparative analysis of *KANSARL* fusion transcripts between the MULCD and SLCD datasets. MULCD and SLCD represented 20 lung cancer patients from University of Michigan and 168 lung cancer samples from South Korean Genomic Medicine Institute. Black and green squares represented total numbers of samples and the numbers of *KANSARL*-positive samples. Red cylinders indicated the percentages of *KANSARL*-positive samples.

Supporting Table 2: Comparison of differences of *KANSARL+* samples between MULCD and SKLCD samples

Types	# of Samples	# of *KANSARL+*	% of *KANSARL+*	Z Scores	Probabilities(p)
**MULCD**	20	8	40	8.37775	< 0.00001
**SKLCD**	168	0	0		

Supporting Table 3: Comparison of number differences of *KANSARL+* samples between HIBCD and SKBCP samples

Types	# of Samples	# of *KANSARL+*	% of *KANSARL+*	Z Scores	Probability(p)
**HIBCD**	163	49	30.06	5.43	< 0.00001
**SKBCP**	78	0	0		

of *KANSARL* fusion transcripts between MULCD and SKLCD is significant (z=8.38, p<0.0005), suggesting that KANSARL fusion transcripts are associated with MULCD lung cancer patients.

Comparative analysis of breast cancer RNA-seq data from USA and South Korea

Breast cancer is the most common incident form of cancer in women around the world and about 1 in 8 (12%) women in the US would develop invasive breast cancer during their lifetime. To investigate whether *KANSARL* fusion transcripts are expressed in breast cancer, we have performed analyses on the breast cancer dataset from USA Hudson Alpha Institute for Biotechnology (designated as HIBCD), which consists of 28 breast cancer cell lines, 42 ER+ breast cancer primary tumors, 30 uninvolved breast tissues adjacent to ER+ primary tumors, 42 triple negative breast cancer (TNBC) primary tumors, 21 uninvolved breast tissues adjacent to TNBC primary tumors and 5 normal breast tissues [9], and breast cancer samples from South Korean (designated as SKBCP), which have samples from 22 HRM (high-risk for distant metastasis) and 56 LRM (low-risk for distant metastasis) breast cancer patients (PRJEB9083 2015). Supporting Figure 3 shows that 50 (or about 30%) HIBCD breast samples have been found to have *KANSARL* fusion transcripts while no SKBCP patients have been observed to have *KANSARL* fusion transcripts. Supporting Table 3 shows that the difference between HIBCD and SKBCP has been shown by $\chi 2$-test to be statistically significant ($p \leq 0.001$), suggesting that breast cancer patients from South Korea had no *KANSARL fusion* transcripts.

Since HIBCD had multiple breast cancer types, we have performed further data analysis of the HIBCD breast samples. Supporting Figure 4 and Supporting Table 4 show that normal tissues, breast cancer cell lines, TNBC primary tumors and uninvolved breast tissues adjacent to TNBC primary tumors have 23.8% to 28.5% of *KANSARL*-positive samples while ER+ breast cancer

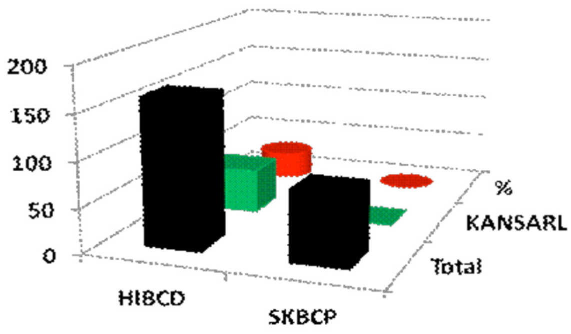

Supporting Figure 3: Comparative analysis of KANSARL fusion transcripts between the HIBCD and SKBCD datasets. HIBCD and SKBCD represent 163 breast cancer samples from Hudson Alpha Institute for Biotechnology and 78 breast cancer patients from South Korean, respectively. Black and green squares represent total numbers of samples and the numbers of KANSARL-positive samples. Red cylinders indicate the percentages of KANSARL-positive samples.

Supporting Table 4: Comparison of number differences of *KANSARL*+ samples among different subtypes of breast cancers in HIBCD samples

Types	# of Samples	# of *KANSARL+*	% of *KANSARL+*	Z Scores	Probabilities(p)
**ER+**	42	15	35.7	-0.39	0.70
**ER+BTA**	30	12	40.0	0.88	0.38
**Normal**	5	1	20.0	-0.19	0.85
**TNBC**	42	10	23.8	-0.42	0.50
**TNBCBTA**	21	6	28.6	0.28	0.78
**BCCL**	28	7	25.0		

Supporting Table 5: Comparison of number differences of *KANSARL*+ samples among the NLD, BCLD, YLD and ULD samples

Types	# of Samples	# of KANSARL+	% of KANSARL+	Z Scores	Probabilities(p)
**NLD**	41	15	36.59	3.11	0.002
**BCLD**	23	8	34.78	3.23	0.001
**YLD**	13	5	38.46	3.01	0.003
**ULD**	20	0	0		

primary tumors and uninvolved breast tissues adjacent to ER+ primary tumor are 35.7% and 40% *KANSARL*-positive percentages of the TNBC samples are much closer to the normal one, which are shown to have no statistical differences. On the other hand, the *KANSARL*-positive ratios in the ER+ samples are 15% higher than the normal one, suggesting that *KANSARL* fusion transcripts have much bigger impacts on ER+ breast cancer than TNBC breast cancer.

Comparative analysis of lymphoma RNA-seq data from North America and Uganda

To investigate whether the *KANSARL* fusion transcripts were expressed in cancer samples from the African population, we have analyzed the Uganda lymphomas dataset (designated as ULD), which contains 20 lymphoma samples [11]. We have performed analyses of multiple lymphoma RNA-seq

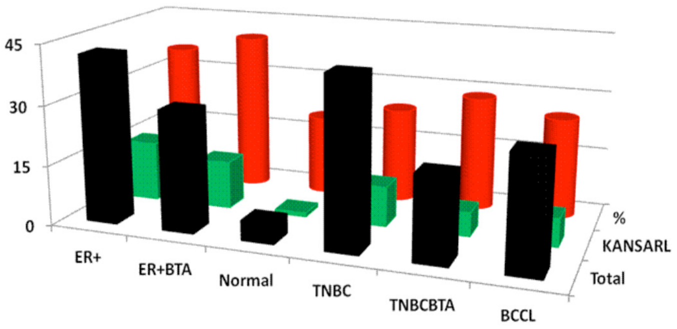

Supporting Figure 4: Characterization of HIBCD breast datasets. Black and gray bars indicated total numbers of samples and numbers of samples having *KANSARL* fusion transcripts, respectively. Dark gray cylinders were percentages of *KANSARL*-positive samples in the datasets. Black and green squares represented total numbers of samples and the numbers of *KANSARL*-positive samples. Red cylinders indicate the percentages of *KANSARL*-positive samples.

datasets including NCI lymphoma dataset (designated as NLD), which has 28 sporadic form Burkitt Lymphoma (BL) patient biopsy samples and 13 BL cell lines [12], Yale University T-cell lymphoma dataset (designated as YLD), which has 13 cutaneous T cell lymphoma and BC Cancer Agency lymphoma data (designated as BLD), in which 23 RNA-seq data of diffuse large B-cell lymphoma have been identified [14]. Even though lymphoma subtypes and the sample sizes are different, we have found that 34% to 38% of NLD, YLD and BLD samples had *KANSARL* fusion transcripts (Supporting Figure 5). On the other hand, no single copy of *KANSARL* fusion transcripts have been detected in 20 ULD lymphoma samples (Supporting Figure 5). Supporting Table 5 shows that the differences of *KANSARL*-positive samples between Uganda and North America are statistically significant ($Z \geq 3.0$; $p \leq 0.0026$) and suggested that Uganda lymphomas are not associated with *KANSARL* fusion transcripts.

In summary, Supporting Figures 1~5 and Supporting Tables 1~5 show that samples of diverse types of cancer from North America (USA and Canada) have been found to have highly recurrent *KANSARL* fusion transcripts, which range from 30% in breast cancer to 52% in prostate tumors. In contrast, *KANSARL* fusion transcripts had been detected in two glioblastoma samples from China and Hela-3 cancer cell line, ethnicity of which was black. No *KANSARL* fusion transcripts have been found in the rest of the cancer samples from South Korea, China and Uganda. Based on localities of health services, we can conclude that *KANSARL* fusion transcripts have been rarely found in the cancer samples from Asian and African ancestry origins and are specifically associated with cancer samples of European ancestry origin.

MATERIALS AND METHODS

Materials

RNA-seq data

Raw RNA-seq data were downloaded from NCBI, ENCODE and ENA, respectively. The human genomic DNA sequences (hg19) were downloaded from UCSC (http://hgdownload.soe.ucsc.edu/downloads.html). The human mRNA/EST dataset was downloaded from Aceview (https://www.ncbi.nlm.nih.gov/ieb/research/acembly/Download/Downloads.html).

Cell lines

Lymphoma cell DHL-4, DHL-5, DHL-8, DHL-10, OCI-Ly10 and Val, ly-10 and Ferage were purchased from ATCC (Manassas, VA).

Methods

Cell cultures

The cell lines were cultured at 37°C at ATCC-formulated RPMI-1640 Medium. To maintain the cell

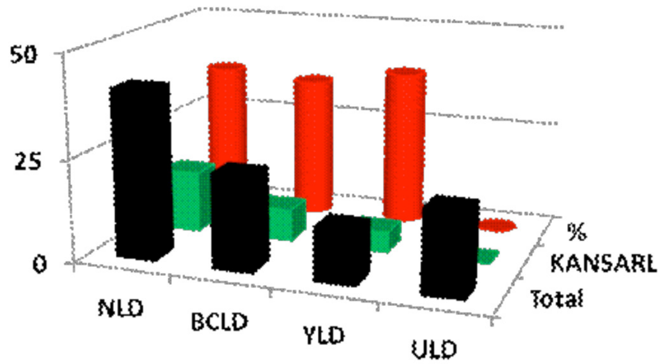

Supporting Figure 5: Comparative analysis of *KANSARL* fusion transcripts among the NLD, BCLD, YLD and ULD datasets. NLD, BCLD, YLD and ULD represented 41 sporadic forms Burkitt Lymphoma from National Cancer Institute, 13 cutaneous T cell lymphoma from Yale University, 23 diffuse large B-cell lymphoma from BC Cancer Agency, and 20 lymphoma samples from Uganda. Black and gray bars indicated total numbers of samples and numbers of samples having *KANSARL* fusion transcripts, respectively. Black and green squares represented total numbers of samples and the numbers of *KANSARL*-positive samples. Red cylinders indicate the percentages of *KANSARL*-positive samples.

lines, the media were changed in every 2 to 3 days (depending on cell density).

Identification of fusion transcripts by SCIF (SplicingCodes Identify Fusion Transcripts)

To use human splicingcodes to identify human fusion transcripts, a human splicingcode table was generated as described previously [15, 16]. Briefly, a file of Aceview.ncbi_37.genes was downloaded from Aceview (https://www.ncbi.nlm.nih.gov/ieb/research/acembly/Download/Downloads.html). The reasons that we selected Aceview genes were that majority of their human gene models have been among the most accurate ones based on our testing results and they have read-through fusion transcripts. The first step was to remove highly-repetitive sequences, poor quality sequences, and duplica ted sequences. Our collection of introns from 382,279 was reduced to 230,000, the majority of which are unique. Splice sites were divided into 5' and 3' splice sites. Then 5' splice site was divided into 5' exonic sequences and 5' intronic sequences and 3' splice site was divided into 3' intronic sequences and 3' exonic sequences to generate a splicingcodes table.

Supplementary Figure 1a showed a diagram of using the human splicingcodes to identify fusion transcripts from human RNA-seq data. As shown in Supplementary Figure 1a, from the first nucleotides of an RNA-seq read, we took the 20 bp sequences as a key, which was searched in the splicingcodes until an identical sequence was found. Then, we took 3' 20bp sequences immediately downstream of this identical sequence to search the splcingcode table. If both sequences were in the splicingcode table and 5' and 3' exonic keys were from different genes, this sequence read are putative fusion transcript candidate for further validation. To improve the alignment accuracy and speed, we have added an important step to continue sequence alignments. As shown Supplementary Figure 1b, the sequences upstream of the 5' key and downstream of the 3' key of the RNA-seq read were further aligned to the corresponding genomic region of 5' and 3' exons. If both regions are identical to the corresponding genomic regions, this read was then blasted against AceView human mRNAs/EST and the human gene databases containing 20-kb upstream sequences of 5' genes and 20-kb downstream sequences of 3' genes, to remove sequences from pseudogenes, gene duplications and alternatively-spliced sequences.

Classification of *KANSARL* fusion transcripts in a sample

After total fusion transcripts were generated by SCIF from an RNA-seq dataset, the false positive fusion transcripts were removed by blast and manual inspections. We then checked whether a sample had six KANSARL fusion isoforms. After we confirmed that *KANSARL* is a familially-inherited fusion gene, all samples were re-analyzed and re-classified. If a sample had one or more copies of *KANSARL* fusion transcripts, this sample were thought to be *KANSARL*-positive. If a sample didn't have any *KANSARL* fusion transcript, this sample were thought to be *KANSARL*-negative.

Identification of the genomic breakpoints of *KANSARL* fusion gene

To identify the genomic breakpoint of *KANSARL* fusion gene, we have downloaded the *KANSL1* and *ARL17A* genomic sequences from Aceview (https://www.ncbi.nlm.nih.gov/IEB/Research/Acembly/index.html), respectively. The *KANSL1* and *ARL17A* genomic sequences are used to generate continuous genomic *KANSL1* and *ARL17A* hash tables of 50 bp keys in length, respectively. That is, from the first nucleotide of the *KANSL1* and *ARL17A* genomic sequences, at every 10bp gap, we continuously take 50 bp sequences as the hash table key. If the keys have multiple copies, only the first one will be used in the hash tables. In order to locate the *KANSARL* genomic breakpoints, we have downloaded WGS data (Accession: PRJEB3381) of grandparents, parents and partial offspring of the CEPH/UTAH family 1463 provided by platinumgenomes.org due to their high genomic sequence qualities, large NGS data sizes and paired-end sequences. (Note: The WGS data of daughters and sons of the CEPH/UTAH family 1463 have recently been transferred into controlled-access dbGap. And their genomic data analysis has been negatively affected). To detect the *KANSARL* genomic breakpoints, a NGS sequence read is divided into two parts from the middle. Starting the first nucleotide of the first part, a 50 bp key sequence are continuously taken to scan the *KANSL1* genomic hash table. If one of the 50 bp sequences of the first part NGS sequence read is in the *KANSL1* genomic hash table, then we check whether one of the 50 bp sequences of the second part NGS sequence read can be found in the ARL17A genomic hash table. If the 50 bp sequences from the first part and the second part of the NGS sequence read are found in both *KANSL1* and *ARL17A* genomic hash tables, this NGS sequence read contains a putative KANSARL genomic breakpoint. Then, Human Blast search (https://genome.ucsc.edu/cgi-bin/hgBlat?command=start) is used to confirm whether the NGS sequence read has both correct *KANSL1* and *ARL17A* genomic sequences. Multiple copies of such NGS sequence reads from breakpoint regions of the *KANSL1* and *ARL17A* genomic sequences are required to confirm the *KANSARL* genomic breakpoint.

Calculation of relative quantification of *KANSARL* fusion transcripts

To calculate the relative quantification of KANSARL fusion transcripts, GAPDH gene was used

as internal controls. The following formula was used to estimate the relative ratios of quantification of *KANSARL* isoform 1 and 2:

$$R = 2^{-[\Delta CP\ sample\ -\ \Delta CP\ control]}$$

Where CP is the crossing point and is the cycles at which the amplification plot crosses the threshold.

DNA extraction

The Hela-3 total DNA was isolated from a 100-mm culture dish by Qiagen's DNeasy Blood & Tissue Kit as described by the manufacturer.

Z-score test

To compare two different populations, we used the two-tailed Z score analyses to evaluate whether two populations differ significantly on their genetic characteristics. We set the null hypothesis to be that there is no difference between the two population proportions. Z scores were calculated based on the following the formula:

$$Z = \frac{(\bar{p}_1 \quad \bar{p}_2) \quad 0}{\sqrt{\bar{p}(1 \quad \bar{p}) \quad \frac{1}{n_1} + \frac{1}{n_2}}}$$

The Z-scores were calculated at in-silico Project support for life sciences (http://in-silico.net/tools/statistics/ztest).

Fisher exact test

Based on a 2 × 2 contingency table, the SAS PROC FREQ procedure was used to compare two groups with a dichotomous dependent variable. The likelihood ratio chi-square was calculated and then subjected to Fisher's exact test to analyze the difference of the frequencies between two groups. $p<0.05$ was considered statistically significant. The relative risk [11] and 95% confidence intervals (CIs) were estimated. All the analyses were conducted with SAS statistical software, version 9.4 (SAS Institute, Cary, NC, USA).

REFERENCES

1. Li X, Battle A, Karczewski KJ, Zappala Z, Knowles DA, Smith KS, Kukurba KR, Wu E, Simon N, Montgomery SB. Transcriptome sequencing of a large human family identifies the impact of rare noncoding variants. Am J Hum Genet. 2014; 95:245–56.
2. Gill BJ, Pisapia DJ, Malone HR, Goldstein H, Lei L, Sonabend A, Yun J, Samanamud J, Sims JS, Banu M, Dovas A, Teich AF, Sheth SA, et al. MRI-localized biopsies reveal subtype-specific differences in molecular and cellular composition at the margins of glioblastoma. Proc Natl Acad Sci USA. 2014; 111:12550–55.
3. Bao ZS, Chen HM, Yang MY, Zhang CB, Yu K, Ye WL, Hu BQ, Yan W, Zhang W, Akers J, Ramakrishnan V, Li J, Carter B, et al. RNA-seq of 272 gliomas revealed a novel, recurrent PTPRZ1-MET fusion transcript in secondary glioblastomas. Genome Res. 2014; 24:1765–73.
4. Wyatt AW, Mo F, Wang K, McConeghy B, Brahmbhatt S, Jong L, Mitchell DM, Johnston RL, Haegert A, Li E, Liew J, Yeung J, Shrestha R, et al. Heterogeneity in the inter-tumor transcriptome of high risk prostate cancer. Genome Biol. 2014; 15:426.
5. Ren S, Peng Z, Mao JH, Yu Y, Yin C, Gao X, Cui Z, Zhang J, Yi K, Xu W, Chen C, Wang F, Guo X, et al. RNA-seq analysis of prostate cancer in the Chinese population identifies recurrent gene fusions, cancer-associated long noncoding RNAs and aberrant alternative splicings. Cell Res. 2012; 22:806–21.
6. Ju YS, Lee WC, Shin JY, Lee S, Bleazard T, Won JK, Kim YT, Kim JI, Kang JH, Seo JS. A transforming KIF5B and RET gene fusion in lung adenocarcinoma revealed from whole-genome and transcriptome sequencing. Genome Res. 2012; 22:436–45.
7. Seo JS, Ju YS, Lee WC, Shin JY, Lee JK, Bleazard T, Lee J, Jung YJ, Kim JO, Shin JY, Yu SB, Kim J, Lee ER, et al. The transcriptional landscape and mutational profile of lung adenocarcinoma. Genome Res. 2012; 22:2109–19.
8. Balbin OA, Malik R, Dhanasekaran SM, Prensner JR, Cao X, Wu YM, Robinson D, Wang R, Chen G, Beer DG, Nesvizhskii AI, Chinnaiyan AM. The landscape of antisense gene expression in human cancers. Genome Res. 2015; 25:1068–79.
9. Varley KE, Gertz J, Roberts BS, Davis NS, Bowling KM, Kirby MK, Nesmith AS, Oliver PG, Grizzle WE, Forero A, Buchsbaum DJ, LoBuglio AF, Myers RM. Recurrent read-through fusion transcripts in breast cancer. Breast Cancer Res Treat. 2014; 146:287–97.
10. Lee JH, Zhao XM, Yoon I, Lee JY, Kwon NH, Wang YY, Lee KM, Lee MJ, Kim J, Moon HG, In Y, Hao JK, Park KM, et al. Integrative analysis of mutational and transcriptional profiles reveals driver mutations of metastatic breast cancers. Cell Discov. 2016; 2:16025.
11. Abate F, Ambrosio MR, Mundo L, Laginestra MA, Fuligni F, Rossi M, Zairis S, Gazaneo S, De Falco G, Lazzi S, Bellan C, Rocca BJ, Amato T, et al. Distinct Viral and Mutational Spectrum of Endemic Burkitt Lymphoma. PLoS Pathog. 2015; 11:e1005158.
12. Schmitz R, Young RM, Ceribelli M, Jhavar S, Xiao W, Zhang M, Wright G, Shaffer AL, Hodson DJ, Buras E, Liu X, Powell J, Yang Y, et al. Burkitt lymphoma pathogenesis and therapeutic targets from structural and functional genomics. Nature. 2012; 490:116–20.
13. Choi J, Goh G, Walradt T, Hong BS, Bunick CG, Chen K, Bjornson RD, Maman Y, Wang T, Tordoff J, Carlson K,

SUPPLEMENTARY FIGURES AND TABLES

Overton JD, Liu KJ, et al. Genomic landscape of cutaneous T cell lymphoma. Nat Genet. 2015; 47:1011–19.

14. Morin RD, Mungall K, Pleasance E, Mungall AJ, Goya R, Huff RD, Scott DW, Ding J, Roth A, Chiu R, Corbett RD, Chan FC, Mendez-Lago M, et al. Mutational and structural analysis of diffuse large B-cell lymphoma using whole-genome sequencing. Blood. 2013; 122:1256–65.
15. Zhuo DC, Zhu S, Dong C, Glass AD. Decipering splicing codes of spliceosomal introns BIOCOMP 2012. Las Vagas, Nevada, USA: CSREA Press; 2012. pp. 521–7.
16. Zhuo D, Madden R, Elela SA, Chabot B. Modern origin of numerous alternatively spliced human introns from tandem arrays. Proc Natl Acad Sci USA. 2007; 104:882–86.

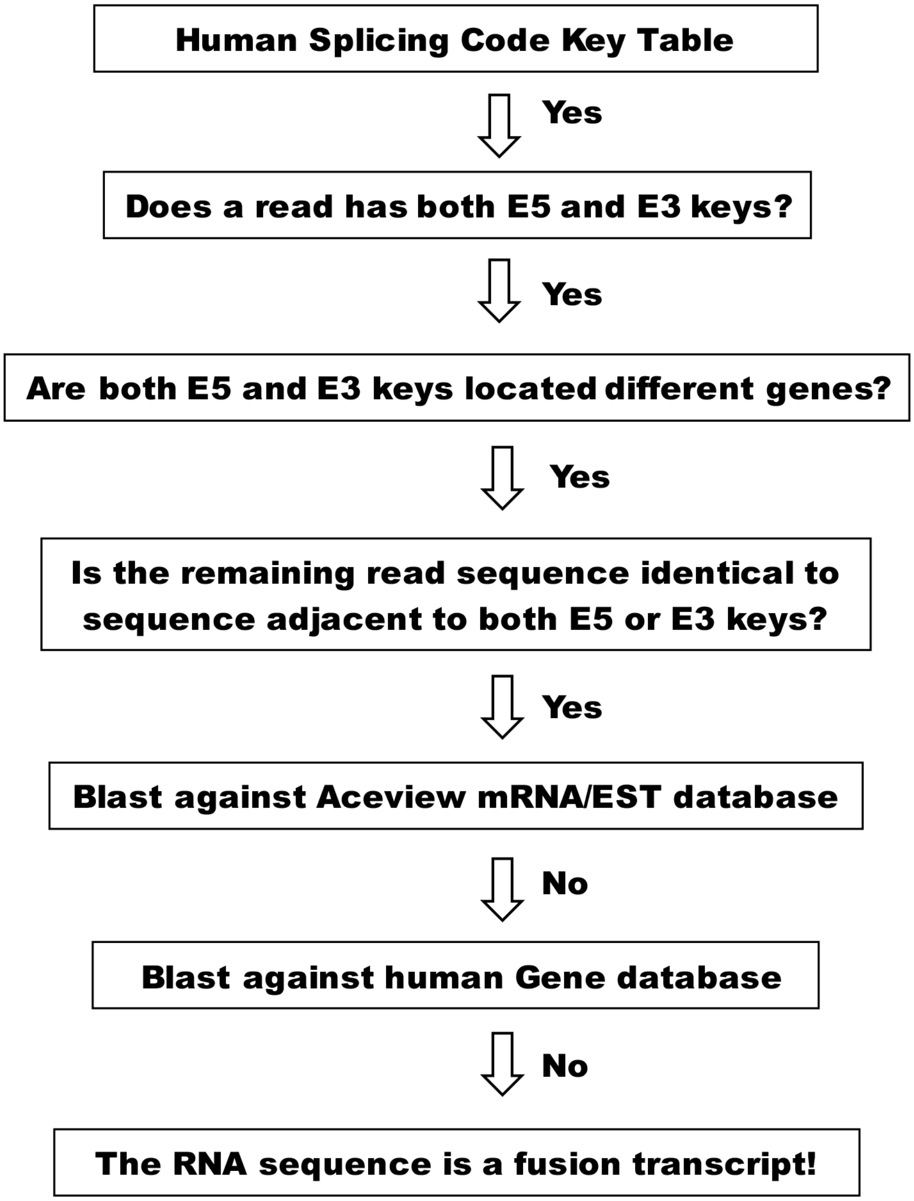

Supplementary Figure 1: Flowchart of SCIF (SplicingCodes Identify Fusion Transcripts). Each step of the SCIF program has results of "Yes" or "No". "Yes" means a positive result while ""No" means a negative result. If each step in SCIF has an opposite result indicated by the arrow, SCIF considers this RNA-seq read as a sequence from one gene.

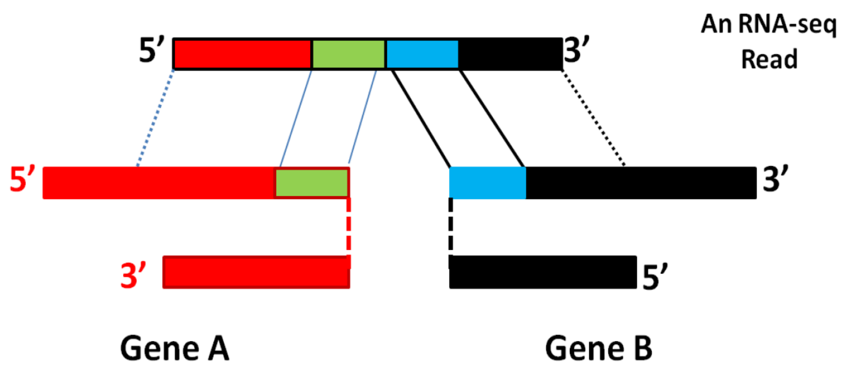

Supplementary Figure 2: Diagram of an RNA-seq sequence read for alignment of both 5' and 3' genes. The red and block squares represent Gene A (5' Gene) and Gene B (3' Gene), respectively. The green and blue squares indicate identical key sequences of genes A and B, respectively. The blue solid and dash lines indicate identical 5' key sequence and 5' adjacent region of Gene A, respectively. The black solid and dash lines represent identical 3' key sequence and 3' adjacent region of Gene B, respectively. The red and black dashed lines represent the introns of Gene A and Gene B, respectively.

A

```
gctgatcccgagcagcgtcatgtacccctgtttctgtgtggagacagtagaatataaaaa
 A  D  P  E  Q  R  H  V  P  L  F  L  C  G  D  S  R  I
taacaccttcgctgtctgggatgttggcagccacttcaaaatcagacctctgtggcagca
```

B

```
tatcgaattcgtcagcaaacagacatttacaaacagatacgtgctaataaggtttctgtg
 Y  R  I  R  Q  Q  T  D  I  Y  K  Q  I  R  A  N  K  V  S  V
tggagacagtagaatataaaaataacaccttcgctgtctgggatgttggcagccacttca
 W  R  Q  -
```

C

```
tatcgaattcgtcagcaaacagacatttacaaacagatacgtgctaataaggtgccagaa
 Y  R  I  R  Q  Q  T  D  I  Y  K  Q  I  R  A  N  K  V  P  E
gcccaggaagcacacatcaaggctcacttgccagcggggtgctgccaataaaatgtagtc
 A  Q  E  A  H  I  K  A  H  L  P  A  G  C  C  Q  -
```

D

```
tatcgaattcgtcagcaaacagacatttacaaacagatacgtgctaataagagctatcag
 Y  R  I  R  Q  Q  T  D  I  Y  K  Q  I  R  A  N  K  S  Y  Q
cttcccagtttgcacaattcatcaagaaattatgcggggtcaccggcacaaatgatgagg
 L  P  S  L  H  N  S  S  R  N  Y  A  G  S  P  A  Q  M  M  R
catctcctggaagcttaacttcttatccatcccatctcttggacagatgatgccagttaa
 H  L  L  E  A  -
```

E

```
gctgatcccgagcagcgtcatgtacccctagctatcagcttcccagtttgcacaattcat
 A  D  P  E  Q  R  H  V  P  L  A  I  S  F  P  V  C  T  I  H
caagaaattatgcggggtcaccggcacaaatgatgaggcatctcctggaagcttaacttc
 Q  E  I  M  R  G  H  R  H  K  -
```

F

```
aagcggaggcttgttcgacccaacagcatcgttcctctttccaagaaggttcgtgagcca
 K  R  R  L  V  R  P  N  S  I  V  P  L  S  K  K  V  R  E  P
cacatctccccacaccaagctcctccatacaagacctcggactgcatcacgtaaatgctt
 H  I  S  P  H  Q  A  P  P  Y  K  T  S  D  C  I  T  -
tttcaggggcaaaatctagagaatctgaaatggtgagccttttttcctt
```

Supplementary Figure 3: Puative truncated protein sequences of six *KANSARL* isoforms. **(A)** isoform 1; **(B)** isoform 2; **(C)** isoform 3; **(D)** isoform 4; **(E)** isoform 5; and **(F)** isoform 6; Underlined sequences are from *ARL17A* gene.

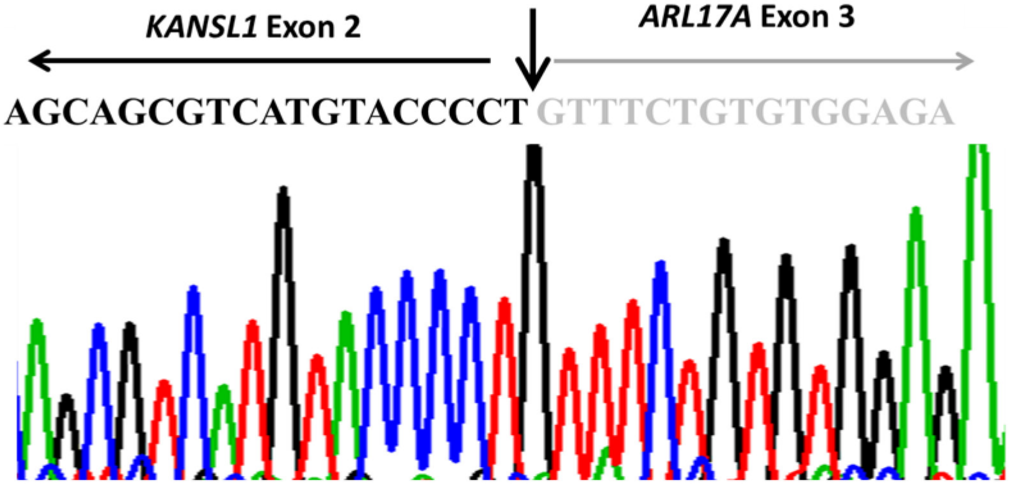

Supplementary Figure 4: Validation of the fusion junction of the *KANSARL* isoform 1 by DNA sequencing. Sanger sequencing validates fusion junction sequences of *KANSARL* isoform 1 PCR products purified from agarose gel in Figure 1C. The black and gray letters represent 5’ and 3’ exonic sequences, respectively. Horizontal arrows represent *KANSL1* exon 2 and *ARL17A* exon 3. Vertical arrow indicates the fusion junctions of *KANSARL* isoform 1.

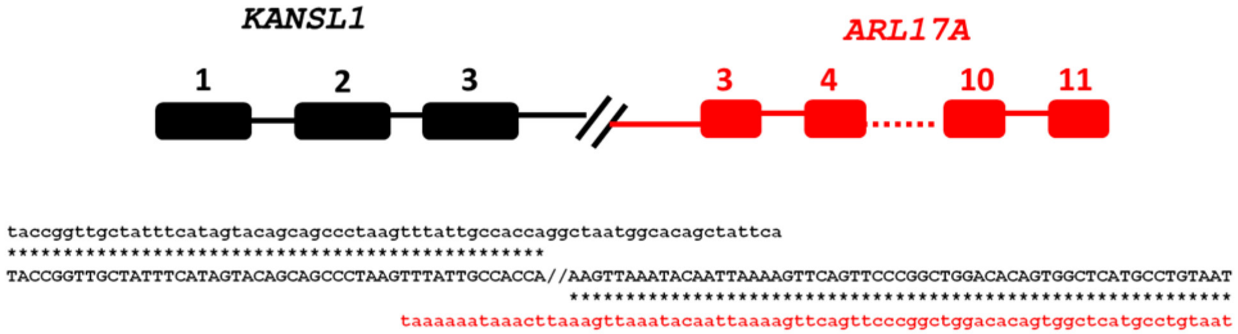

Supplementary Figure 5: Diagram of the genomic breakpoint 1 of *KANSARL* fusion gene detected by analysis of paired-end WGS sequences in the CEPH/Utah Pedigree 1463. The capital letters indicate a sequence read. Two large and small forward slashes represent genomic breakpoint 1 of *KANSL1* and *ARL17A* gene and insert gaps between the paired-end sequence, respectively. Black and red horizontal lines represent *KANSL1* and *ARL17A* introns, respectively. Small black and red letters are *KANSL1* and *ARL17A* intron sequences, respectively. Stars indicate that the regions are identical regions between a sequence read and *KANSL1* or *ARL17A* intron sequences. Red dashed line is omitted sequences. The numbers above the black and red boxes indicate the exon numbers. Analysis of the sequences shows that the genomic breakpoint 1 of *KANSARL* fusion gene is located between *KANSL1* intron 3 and *ARL17A* intron 2. The sequences from these *KANSL1* and *ARL17A* regions have been used to design PCR primers for amplification of genomic breakpoint 1 of *KANSARL* fusion gene.

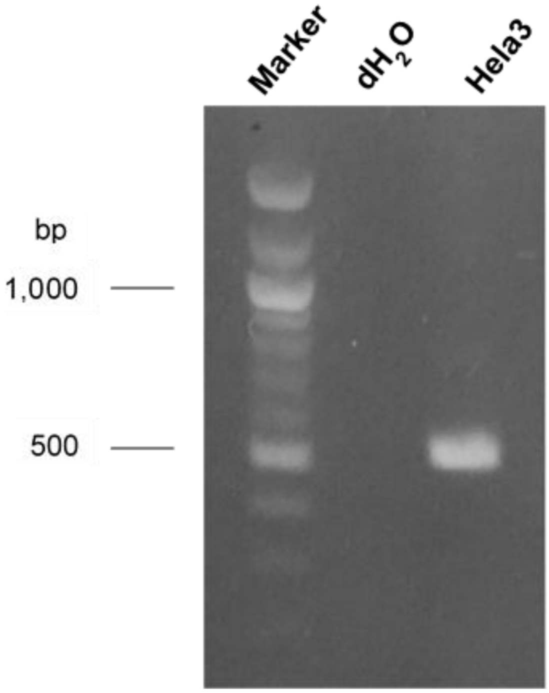

Supplementary Figure 6: PCR amplification of KANSARL genomic breakpoint. Total DNAs were isolated from Hela-3 cells by Qiagene's DNeasy Kit and were used to perform genomic amplification using the primers 5'-TCATCCACAGAGGAGTCACTTAGG-3' (forward) and 5'-AAGTTCAGTTCCCGGCTGG-3' (reverse). The PCR product was separated on 1.2% agarose gel. DNA Markers are 100bp markers from NEB.

GGCGTGCATGTAACTGTGACCCATTCCATGTTCCTTCGGTCCTGTGTAAAG

CAGAAGCTTGCTTCACTCTTGGAGTTTTCTAGGCCTGTGCAGCCTAAGCAT

GATCCTGGCTCTGACTCGGGACATGGCGAAGGAAAAGGAGACTCTTGACC

TTTTTCTTCGGCTTCTACCGGTTGCTATTTCATAGTACAGCAGCCCTAAGTTT

ATTGCCACCAGGCTAATGGCACAGCTATTCAACACTGACCCAGTTTAAACC

TGGAATGTTAGGGAAGCCCATGTTGGGGTAGCATGACAG//*CTGAGAAGCTG*

GGATTACAGGTGCACGCCACCACACCCGGCTAATTTTTGTATTATTAGTGGA

GACGGAGTTTTACCCTGTTGGCCAGGCTGGTCTCGAACTCCTGACCTCGGG

Supplementary Figure 7: Sequencing validation of the genomic breakpoint in the *KANSARL* fusion gene. The genomic PCR product was sequenced directly using the PCR forward primer 5'-TCATCCACAGAGGAGTCACTTAGG-3'. The black and blue letters indicate *KANSL1* and *ARL17A* intronic sequences, respectively. Two forward slash lines represent the *KANSARL* fusion genomic breakpoint.

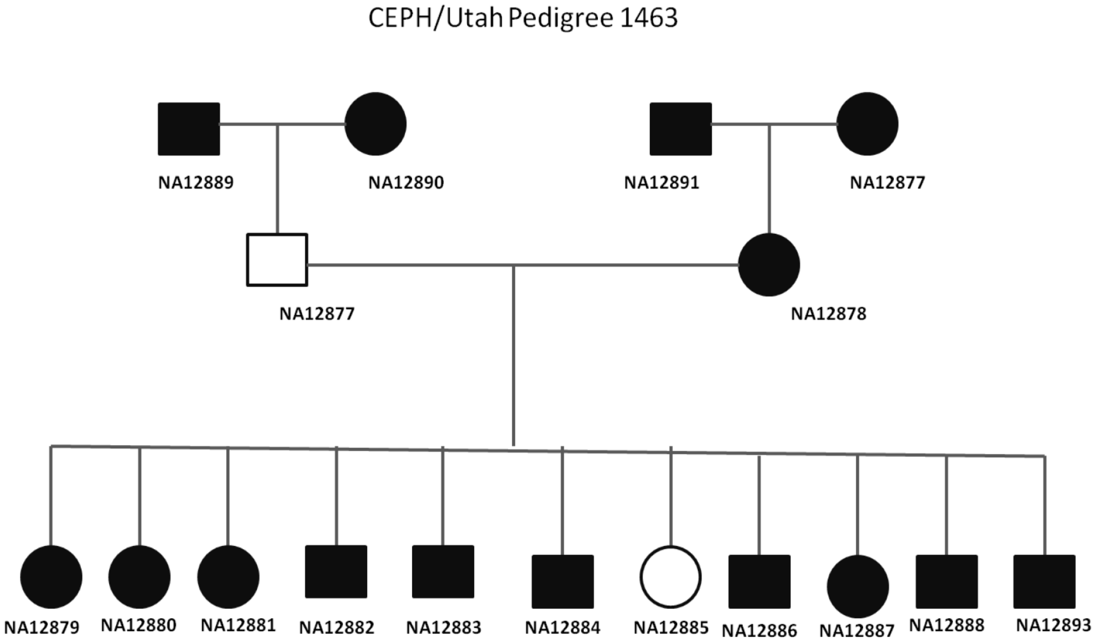

Supplementary Figure 8: Diagram of correct *KANSARL* familial-inheritance of the CEPH/Utah Pedigree 1463. The diagram of familial inheritance of the CEPH/Utah Pedigree 1463 was deduced from analysis of *KANSARL* fusion transcripts based on the RNA-seq data [1]. Black and white squares represent *KANSARL*-positive and *KANSARL*-negative males; while black and white squares indicate *KANSARL*-positive and *KANSARL*-negative females. The black lines represent relationships among the family members.

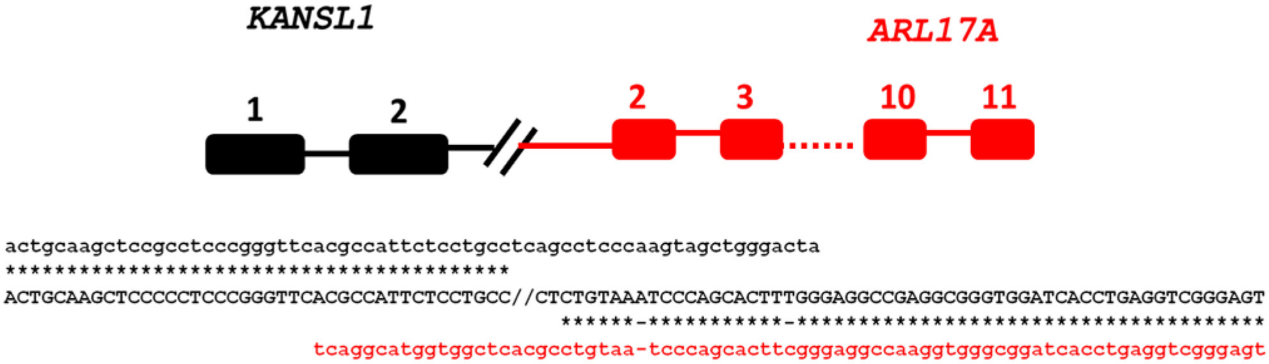

Supplementary Figure 9: Diagram of the genomic breakpoint 2 of *KANSARL* fusion gene detected by analysis of paired-end WGS sequences in the CEPH/Utah Pedigree 1463. The capital letters indicate a sequence read. Two large and small forward slashes represent genomic breakpoint 2 of *KANSL1* and *ARL17A* gene and insert gaps between the paired-end sequences, respectively. Black and red horizontal lines represent *KANSL1* and *ARL17A* introns, respectively. Small black and red letters are *KANSL1* and *ARL17A* intron sequences, respectively. Stars indicate that the regions are identical regions between a sequence read and *KANSL1* or *ARL17A* intron sequences. Red dashed line is omitted sequences. The numbers above the black and red boxes indicate the exon numbers. Analysis of the sequences shows that the genomic breakpoint 2 of *KANSARL* fusion gene is located between *KANSL1* intron 2 and *ARL17A* intron 1.

Supplementary Table 1: Raw accounts and relative ratios of six *KANSARL* isoforms in the ECD39 dataset

KANSARL Isoforms	Counts	% of Total Expression	Folds
**1**	48	1.93	50.69
**2**	2433	97.87	1
**3**	2	0.08	1216.5
**4**	1	0.04	2433
**5**	1	0.04	2433
**6**	1	0.04	2433

Supplementary Table 2: Characteristics of *KANSARL*+ cell lines in the ECD 39

Cell Lines	Raw Counts	NSJPM	Tissues	Tumors	Sexes	Ages	Ethnic
**A375**	60	0.13	Skin	malignant melanoma	Female	54	Caucasian
**A549**	215	0.11	Lung	Carcinoma	Male	58	Caucasian
**G401**	10	0.03	Kidney	rhabdoid tumor	Male	0.25	Caucasian
**H4**	54	0.14	Brain	Neuroglioma	Male	37	Caucasian
**Hela-3**	124	0.06	cervix	Adenocarcinoma	Female	31	Black
**HT29**	194	0.42	colon	colorectal adenocarcinoma	Female	44	Caucasian
**K562**	796	0.22	Bone Marrow	Leukemia	Female	53	Unknown
**Karpas422**	294	1.01	B cells	non-Hodgkin's lymphoma	Female	73	Unknown
**M059J**	31	0.09	Brain	malignant glioblastoma	Male	33	Unknown
**OCI-Ly7**	5	0.02	B cells	non-Hodgkin's lymphoma	Male	48	Unknown
**SK-N-DZ**	690	0.61	Brain	Neuroblastoma	Female	2	Caucasian

Supplementary Table 3: Real-time quantitative PCR amplification of *KARNSARL* isoforms 1 and 2 from A549, Hela-3 and K562 cell lines

Cell Lines	Gene / Isoforms	Repeat 1	Repeat 2	Repeat 3	Average	SD	Fold
**A549**	*GAPDH*	1	1	1	1	0	
	KANSARL Iso2	0.0035	0.0037	0.0036	0.0036	0.000087	63.7
	KANSARL Iso1	5.7E-05	6E-05	6E-05	6E-05	1.12E-06	
**Hela-3**	*GAPDH*	1	1	1	1	0	
	KANSARL Iso2	0.00288	0.00288	0.00278	0.0028	5.66E-05	76.3
	KANSARL Iso1	3.6E-05	4E-05	4E-05	4E-05	1.36E-06	
**K562**	*GAPDH*	1	1	1	1	0	
	KANSARL Iso2	0.0129	0.0128	0.0128	0.0128	5.132E-05	82.9
	KANSARL Iso1	0.0002	0.0002	0.0002	0.00015	1.07E-06	

Supplementary Table 4: Primers used for PCR amplifications in this study

Primer IDs	Primer Sequences (5'→3')
**KANARLIso1F1**	CAAGCCAAGCAGGTTGAGA
**KANARLIso1R1**	TCTCCACACAGAAACAGGGGTA
**KANARLIso4F1**	TTGTGCAAGCCAAGCAGGTT
**KANARLIso4R1**	TGGGAAGCTGATAGCTAGGGGT
**KANARLIso3F1**	TCAGAATGGAAATGGGCTGCA
**KANARLIso3R1**	TTCCTGGGCTTCTGGCACCTT
**KANARLIso6F1**	AGACGCAGGTCAGAATGGAAAT
**KANARLIso6R1**	AAACTGGGAAGCTGATAGCTCT
**KANARLIso5F1**	TGTCTTGGCAGACCACATTC
**KANARLIso5R1**	GGAAAAAGGCTCACCATTTCA
**KANSARLF1**	GCCTTGAGAA AAGCTGCCAG
**KANSARLR1**	AACATCCCAGACAGCGAAGG
**KANSARLF2**	GAGACGCAGGTCAGAATGGA
**KANSARLR2**	AAATGC TGC CAC AGAGGTCT
**GAPDHF1**	CAAGGTCATCCATGACAACTTTG
**GAPDHR1**	GTCCACCACCCTGTTGCTGTAG
**GAPDHqF1**	GCGACACCCACTCCTCCACCTTT
**GAPDHqR1**	TGCTGTAGCCAAATTCGTTGTCATA
**KANSARLgF1**	TGTGCAGCCTAAGCATGATCCT
**KANSARLgR1**	GACACAGTGGCTCATGCCTGTAAT
**gKANSL1F1**	TCATCCACAGAGGAGTCACTTAGG
**gARL17AR1**	AAGTTCAGTTCCCGGCTGG

Supplementary Table 5: Analysis of *KANSARL*$^+$ differences between glioblastoma and normal samples

	# of Samples	# of *KANSARL*$^+$	% of *KANSARL*$^+$	Fisher's exact test
**Gliomas**	27	14	51.85	0.00996
**Normal**	17	2	11.76	

Supplementary Table 6: Differences of *KANSARL*$^+$ samples between BGD and CGD datasets

Types	# of Samples	# of *KANSARL*+	% of *KANSARL*+	Fisher's exact test
**BGD**	272	2	0.74	< 0.00001
**CGD**	27	14	51.85	

Supplementary Table 7: Relationship between germline *KANSARL* fusion transcripts and somatic *TMPRSS2-ERG* fusion transcripts in the VPD samples

Sample Types	# of Samples	# of *TMPRSS2-ERG+*	% of *TMPRSS2-ERG+*	Fisher's exact test
KANSARL+	13	13	100	p<0.001
KANSARL-	12	2	16.67	

Supplementary Table 8: The *KANSARL*-positive patients have increased the numbers of recurrent epigenetic (read-through) fusion transcripts than the *KANSARL*-negative ones

**a**	**Prostate Cancer**			
	***KANSARL*-positive**		***KANSARL*-negative**	
	**Counts**	**%**	**Counts**	**%**
**Genetic**	5	19.23	5	31.25
**Epigenetic**	21	80.77	11	68.75
**Total**	26		16	
**b**	**Glioblastomas**			
	***KANSARL*-positive**		***KANSARL*-negative**	
	**Counts**	**%**	**Counts**	**%**
**Genetic**	1	5	0	0
**Epigenetic**	19	95	6	100
**Total**	20		6	

"Epigenetic" represents read-through fusion transcripts.
"Genetic" is fusion transcripts from translocations, deletions, insertions, and inversions.

Supplementary Table 9: Familial inheritance of *KANSARL* fusion transcripts in the CEPH/Utah Pedigree 1463

Individual ID	Run ID	MB	*KANSARL+*
**NA12877**	SRR1258217	4670	0
**NA12878**	SRR1258218	3709	10
**NA12879**	SRR1258219	4759	11
**NA12880**	SRR1258220	4523	3
**NA12881**	SRR1258221	3548	7
**NA12887**	SRR1258222	3900	5
**NA12888**	SRR1258223	3141	2
**NA12892**	SRR1258224	3509	7
**NA12893**	SRR1258225	3529	8
**NA12882**	SRR1258226	3801	10
**NA12883**	SRR1258227	2644	3
**NA12884**	SRR1258228	3086	4
**NA12885**	SRR1258229	4242	0
**NA12886**	SRR1258230	3485	11
**NA12889**	SRR1258231	3313	15
**NA12890**	SRR1258232	3145	1
**NA12891**	SRR1258233	3189	5

Supplementary Table 10: The putative genomic breakpoints of the *KANSARL* fusion gene detected in the individuals of the CEPH/Utah Pedigree 1463

Sample ID	Run ID	Relationship	GBP1	GBP2
**NA12891**	ERR194160	Grandfather (M)	Yes	
**NA12892**	ERR194161	Grandmother (M)	Yes	
**NA12889**	ERR194158	Grandfather (F)	No	
**NA12890**	ERR194159	Grandmother (F)	Yes	Yes
**NA12877**	ERR194146	Father	No	
**NA12878**	ERR194147	Mother	Yes	Yes
**NA12882**	ERR194151	Son	Yes	Yes
**NA12883**	ERR194152	Son	Yes	
**NA12886**	ERR194155	Son	Yes	Yes
**NA12888**	ERR194157	Son	Yes	
**NA12893**	ERR194162	Son	Yes	

GBP1 and GBP2 represent putative genomic breakpoint 1 and genomic breakpoint 2 of the *KANSARL* fusion gene, respectively. "Yes" or "No" indicate that an individual has the genomic breakpoint 1 of *KANSARL* fusion gene. Capital letters "M" and "F" represent mother's and father heritages, respectively.

Supplementary Table 11: Comparison of *KANSARL*-bearing frequencies in GBR, FIN, TSI and YRI populations

Sample IDs	# of Samples	# of *KANSARL*	% of *KANSARL*	Z Scores	Probabilities (p)
**GRB**	95	32	33.68	6.024	< 0.00001
**FIN**	95	25	26.32	5.206	< 0.00001
**TSI**	93	25	26.88	5.266	< 0.00001
**YRI**	89	0	0.00		

GBR: British from England and Scotland
TSI: Toscani in Italia
FIN: Finnish in Finland
YRI: Yoruba in Ibadan

Supplementary Table 12: Distribution of *KARSARL* fusion transcripts in human heathy tissues and organs in SSTD

Tissues	*KANSARL*
**adipose tissue**	+
**adrenal gland**	+
**ovary**	+
**appendix**	+
**bladder**	+
**bone marrow**	-
**cerebral cortex**	+
**colon**	+
**duodenum**	+
**endometrium**	+
**esophagus**	+
**fallopian tube**	+
**gall bladder**	+
**heart**	+
**kidney**	-
**liver**	+
**lung**	+
**lymph node**	+
**pancreas**	+
**placenta**	+
**prostate**	+
**rectum**	+
**salivary gland**	+

(*Continued*)

Tissues	*KANSARL*
**skeletal muscle**	+
**skin**	+
**small intestine**	+
**smooth muscle**	-
**spleen**	+
**stomach**	-
**testis**	+
**thyroid**	+
**tonsil**	+

The RNA-seq datasets were downloaded from Science for Life Laboratory, Sweden (designated as SSTD), which were originated from tissue samples of 127 human individuals representing 32 different tissues.
